# Stereocontrolled Self-Assembly of a Helicate-Bridged Cu^I^_12_L_4_ Cage That Emits Circularly Polarized Light

**DOI:** 10.1021/jacs.3c11321

**Published:** 2024-01-22

**Authors:** Huangtianzhi Zhu, Luca Pesce, Rituparno Chowdhury, Weichao Xue, Kai Wu, Tanya K. Ronson, Richard H. Friend, Giovanni M. Pavan, Jonathan R. Nitschke

**Affiliations:** † Yusuf Hamied Department of Chemistry, University of Cambridge, Lensfield Road, Cambridge CB2 1EW, United Kingdom; ‡ Department of Innovative Technologies, University of Applied Sciences and Arts of Southern Switzerland, CH-6962 Lugano-Viganello, Switzerland; § Cavendish Laboratory, University of Cambridge, Cambridge CB3 0HE, United Kingdom; ∥ Department of Applied Science and Techology, Politecnico di Torino, 10129 Torino, Italy

## Abstract

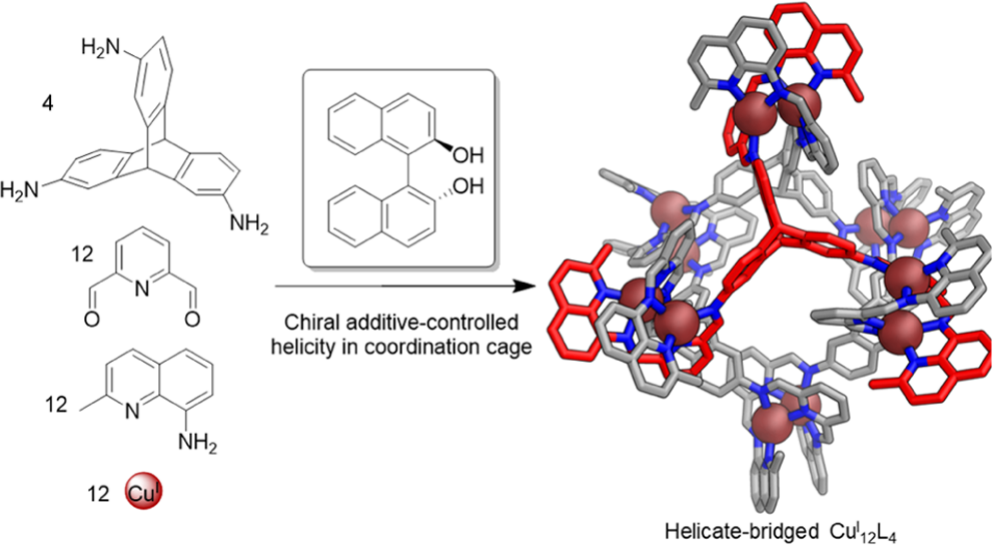

Control over the stereochemistry of metal–organic cages can give rise to useful functions that are entwined with chirality, such as stereoselective guest binding and chiroptical applications. Here, we report a chiral Cu^I^_12_L_4_ pseudo-octahedral cage that self-assembled from condensation of triaminotriptycene, aminoquinaldine, and diformylpyridine subcomponents around Cu^I^ templates. The corners of this cage consist of six head-to-tail dicopper(I) helicates whose helical chirality can be controlled by the addition of enantiopure 1,1′-bi-2-naphthol (BINOL) during the assembly process. Chiroptical and nuclear magnetic resonance (NMR) studies elucidated the process and mechanism of stereochemical information transfer from BINOL to the cage during the assembly process. Initially formed Cu^I^(BINOL)_2_ thus underwent stereoselective ligand exchange during the formation of the chiral helicate corners of the cage, which determined the overall cage stereochemistry. The resulting dicopper(I) helicate corners of the cage were also shown to generate circularly polarized luminescence.

## Introduction

The stereochemical configurations of biomolecules are crucial to the recognition processes that underpin their functions, such as the stereoselective interactions between enzymes and their substrates, and the folding of proteins and nucleic acids into functional higher-order structures.^1−3^ These processes have inspired the design of artificial supramolecular systems where chirality is integral to function, such as metal–organic cages.^4−13^ Enantiopure metal–organic cages enable applications in the fields of chiral recognition, separation, catalysis, and chiroptics,^14−21^ thus extending the functions of achiral and racemic coordination cages.^22−27^ For example, work from the Raymond and Bergmand groups reported an enantiopure [Ga^III^_4_L_6_]^12–^ tetrahedron capable of dynamically resolving racemic ruthenium complexes.^28^ More recently, circularly polarized luminescence (CPL)-active metal–organic assemblies,^29−31^ which hold great potential for chiroptical devices and display technologies, have been reported by the Sun^32^ and Clever^33^ groups. The chirality of those cages frequently originates from the Δ vs Λ handedness of metal vertices, as well as stereocenters incorporated into ligands.^34−39^ We thus anticipated that the incorporation of new chiral moieties, such as chiral Cu^I^ helicates,^40,41^ into cage frameworks could enable the exploration of new functions linked to stereochemistry.^42,43^

Helical supramolecular structures can be prepared by the coordination-driven self-assembly of multitopic ligands around different metal ions. These structures have enabled different functions.^44−48^ Methods to influence the stereochemistry of these assemblies have focused principally upon the use of ligands containing asymmetric centers, with a few notable exceptions, in which chiral information transfers from a guest or solvent to the assembly.^49−58^

A complementary, and rarely employed, strategy involves the use of chiral chelating additives that bind metal ions weakly prior to assembly,^59^ followed by ligand exchange to define the stereochemistry of complexes that incorporate more robustly coordinating ligands. This strategy requires finely tuned binding affinities to secure stereocontrol. Complexation of a chiral additive that binds too strongly renders subsequent ligand exchange infeasible, whereas an additive that binds too weakly is unlikely to affect stereocontrol.

**Figure 1:**
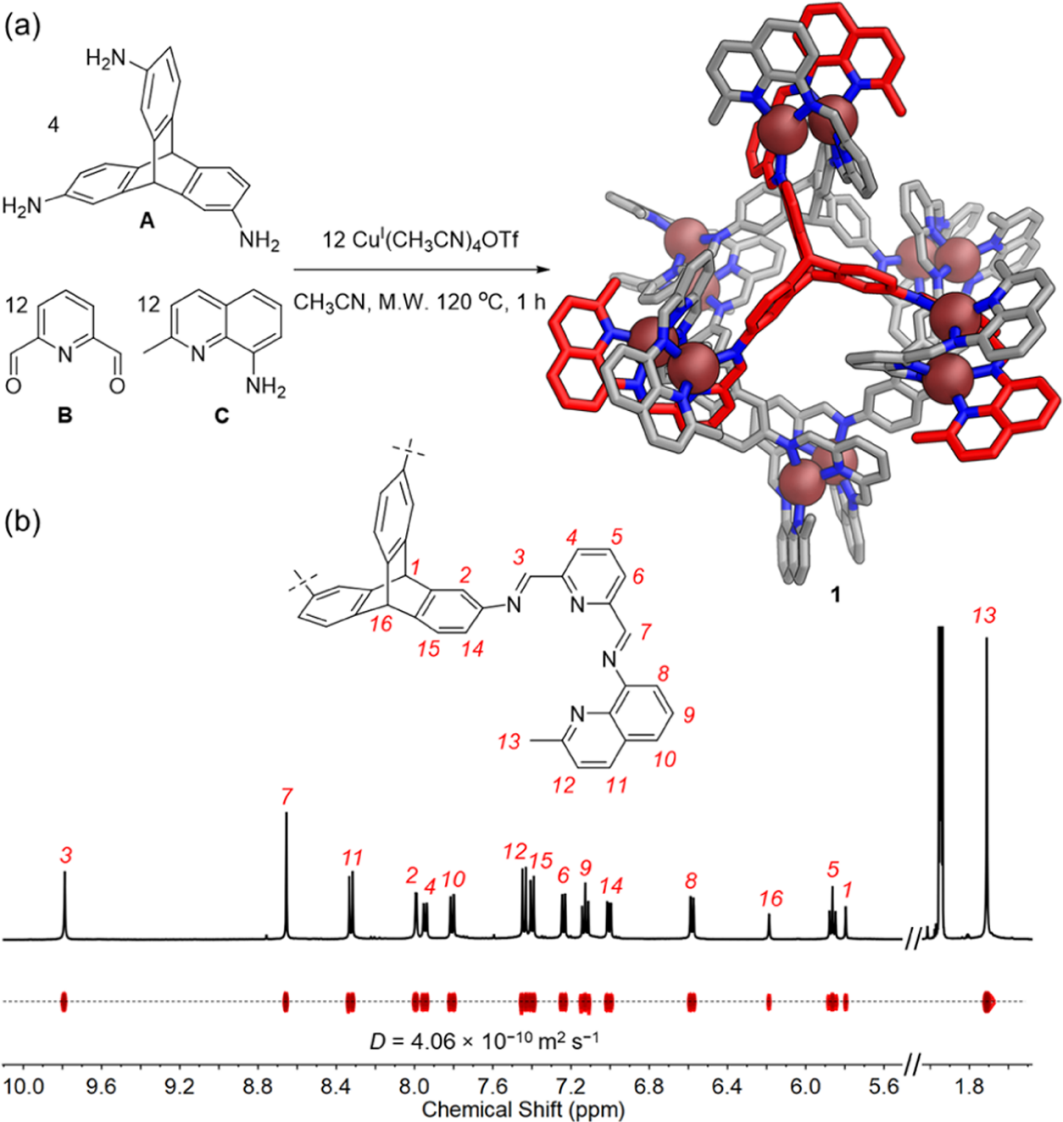
(a) Subcomponent self-assembly of pseudo-octahedral cage **1** with the cage shown as its DFT model. Carbon is shown in gray, nitrogen atoms are blue, copper(I) atoms are burgundy. The carbon atoms of one ligand are shown in red for clarity. (b) Partial chemical structure of the ligand showing NMR peak assignments and ^1^H NMR and DOSY spectra (400 MHz, 298 K, CD_3_CN) of **1**, showing assignments.

Here, we report the self-assembly of a Cu^I^_12_L_4_ cage **1**, the corners of which are composed of dicopper(I) helicates. Prior work has shown that both head-to-tail (HT) and head-to-head (HH) dicopper(I) helicates formed during the subcomponent self-assembly of systems that contain anilines with 2,6-diformylpyridine and 8-aminoquinolines.^60^ In this study, the use of the tris(aniline) 2,7,14-triaminotriptycene **A** (Figure 1) in such systems was found to drive the formation of HT helicates, which formed the six corners of a *T*-symmetric Cu^I^_12_L_4_ pseudo-octahedral cage.

Cage **1** exhibited stereochemical information transfer, whereby the stereochemistry of its helicate corners was governed by the initial binding of 1,1′-bi-2-naphthol (BINOL) to Cu^I^. This weak binding resulted in the formation of a chiral intermediate, which then underwent stereoselective ligand exchange during the formation of the helicate corners of **1** to form cages with exclusively *P* or *M* corner stereoconfigurations. Once formed stereoselectively, **1** did not racemize upon heating to 353 K for 15 days in the absence of BINOL.

Cu^I^ assemblies have potential applications based on their electronic and photophysical features.^61−63^ Following its stereoselective preparation, we found that **1** emitted CPL, potentially enabling its use in the many applications underpinned by circularly polarized luminescence.^29−33^

## Results and Discussion

The reaction of 2,7,14-triaminotriptycene (**A**, 4 equiv), 2,6-diformylpyridine (**B**, 12 equiv), 8-aminoquinaldine (**C**, 12 equiv), and Cu^I^(CH_3_CN)_4_OTf (12 equiv) produced cage **1** as the uniquely observed product (Figure 1a). Electrospray ionization mass spectrometry (ESI-MS) indicated a Cu^I^_12_L_4_ formulation (Figure S8), allowing us to infer that **1** consists of four triptycene moieties and six dicopper(I) helicates. The ^1^H NMR spectrum of **1** contained 16 signals (Figure 1b), with all signals displaying the same diffusion coefficient in the ^1^H diffusion-ordered spectroscopy (DOSY) spectrum. Two-dimensional NMR spectra allowed the assignment of each proton signal of **1** (Figures S4–S7).

The presence of only one set of ligand protons indicated a high-symmetry configuration for **1**, in which all six helicates adopt the same HT or HH conformation. Identical results were also obtained using Cu^I^ salts with different anions, indicating that anions did not exercise a templating effect during cage formation (Figures S10–S16).^64−67^ Changing 8-aminoquinaldine to 8-aminoquinoline, or using 4-bromo-2,6-diformylpyridine in place of the nonbrominated analogue, yielded products with the same Cu^I^_12_L_4_ framework (Figures S17–S20). Following work undertaken by Mastalerz,^8^ we infer the curvature of **A** to have been essential to the formation of **1**, as planar triamines did not generate discrete products (Figure S21).

Despite more than 600 individual attempts to grow single crystals of **1**, none of the crystals obtained diffracted strongly enough to obtain a crystal structure. Density functional theory (DFT) calculations were thus carried out to elucidate the structure of **1**. Different configurations of four triptycene units and six dicopper(I) helicates were combined to form discrete structures. Two conformations of triptycene, denoted “inward” and “outward”, and two helicate configurations, head-to-head and head-to-tail, were considered during structural optimizations. Full details of the structural optimizations undertaken are given in Section 7 in the Supporting Information.

**Figure 2:**
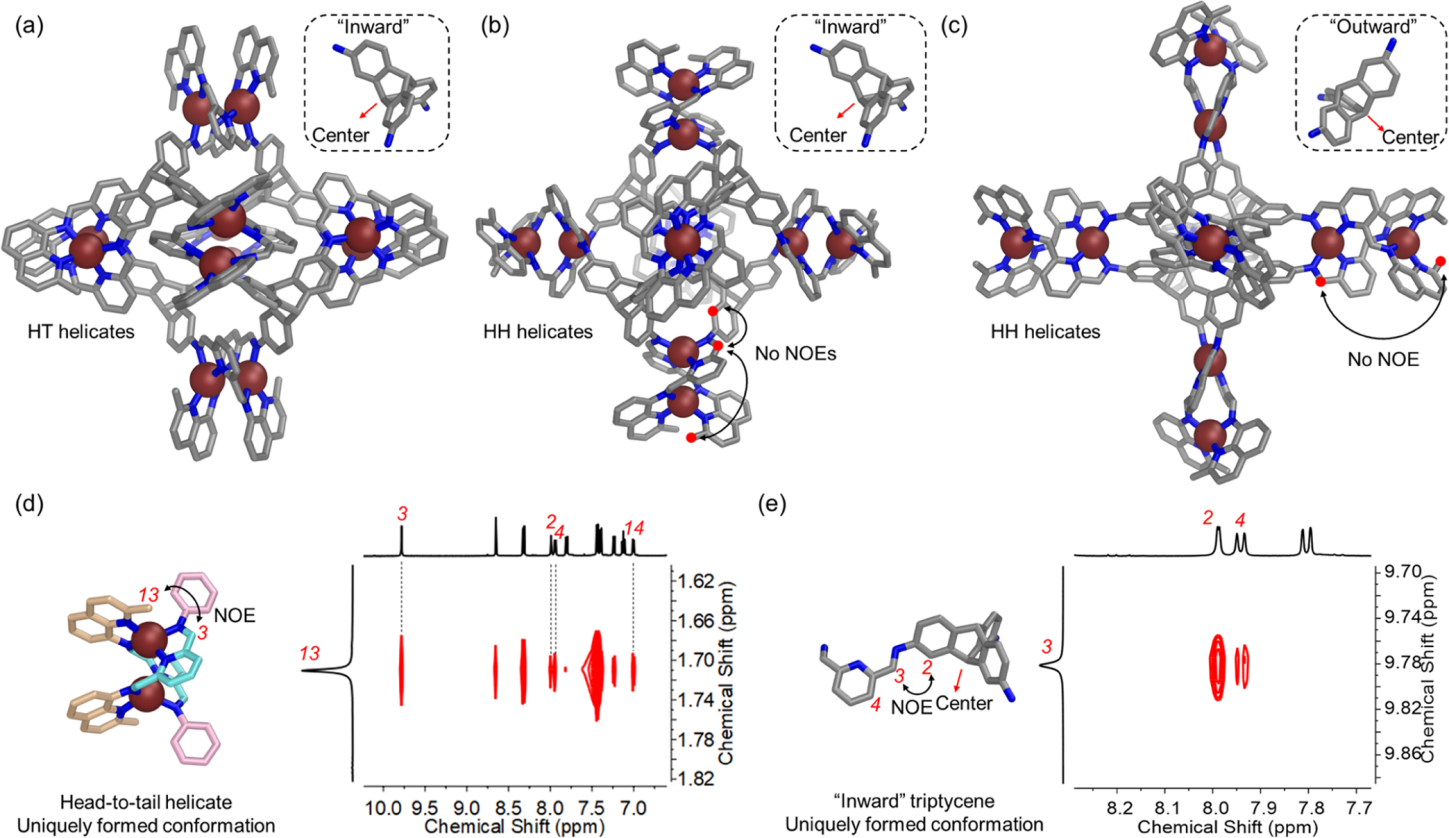
DFT-optimized structures of **1** with (a) “inward”-facing triptycenes and HT helicates, (b) “inward”-facing triptycenes and HH helicates, and (c) “outward”-facing triptycenes and HH helicates. (d, e) Partial NOESY spectra (400 MHz, 298 K, CD_3_CN) of **1** that support the HT conformation and “inward”-facing triptycene configuration, supporting the assignment to **1** of the configuration shown in (a).

Figure 2a–c shows three of the lowest-energy *T*-symmetric frameworks for **1**, each of which is consistent with the ESI-MS and one-dimensional ^1^H NMR data obtained. Only the structure shown in Figure 2a matches with all of our 2D NMR data in two key ways. First, as shown in Figure 2d, nuclear Overhauser effect (NOE) magnetization transfer is observed between methyl proton H_13_ and imine proton H_3_. This observation is consistent with a head-to-tail helicate configuration but not a head-to-head one. Second, the NOE correlation shown in Figure 2e between imine proton H_3_ and triptycene proton H_2_ is consistent with the “inward” triptycene conformation shown, but not an “outward” one. We thus infer **1** to adopt the structure shown in Figure 2a, whose Cu^I^···Cu^I^ distances also match more closely those of dicopper(I) helicates reported than do the other structures considered.^60,68^ The fourth structure, containing head-to-tail helicates and “outward”-facing triptycenes, was eliminated on account of steric hindrance, as shown in Section 7 in the Supporting Information.

**Figure 3:**
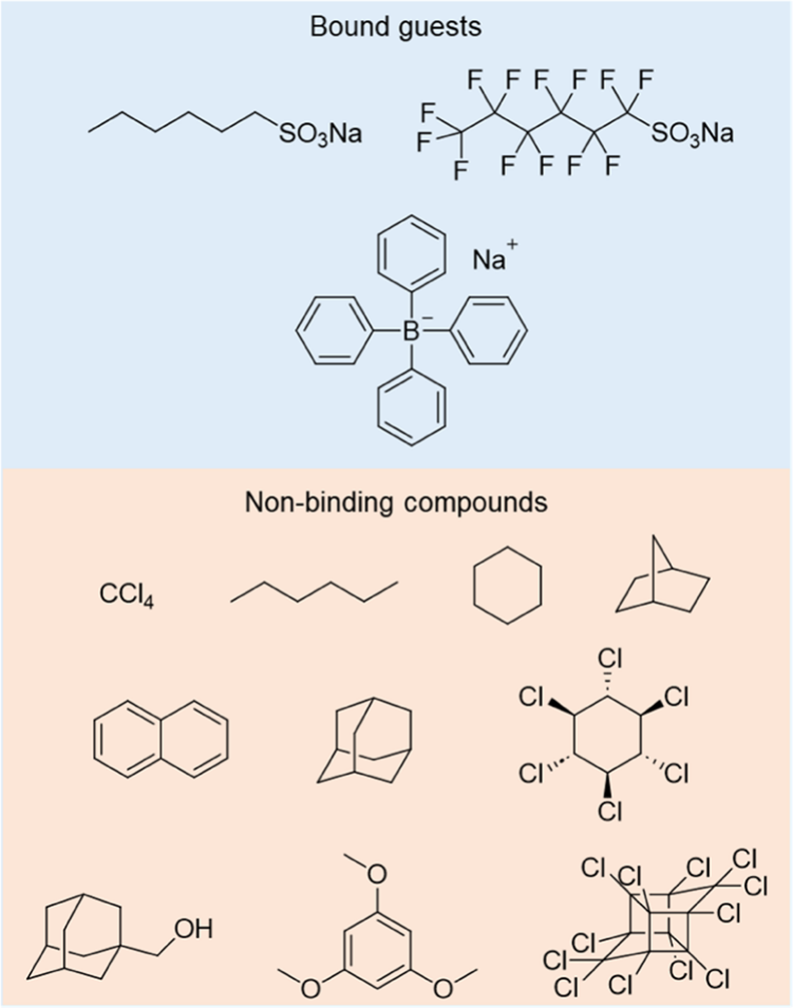
Prospective guests were tested for binding within **1**.

The cavity of the conformation of cage **1** shown in Figure 1a has a volume of 344 Å^3^, calculated using MoloVol (Figure S22).^69^ We were not able to observe neutral guest binding within this cavity, however (Figures 3 and S32). We inferred this lack of binding to be due to large peripheral pores and the lack of stacking interactions between the host and potential guests. To probe the binding ability of the cage toward anionic guests,^70,71^**1** was treated with anions that included organic sulfonates and tetraphenylborate, all of which were complexed by **1** in fast exchange on the NMR time scale. Cage **1** bound 1-hexylsulfonate with a binding constant >10^3^ M^–1^, whereas its complexation of perfluoro-1-hexanesulfonate, a chronic pollutant that has attracted interest due to the need for its environmental remediation,^72^ was weaker, which we attribute to the electron-deficient character of the guest perfluoroalkane chain (Figures S23–S28). We infer that these two guests thread into the cavity of the cage, as reflected in downfield shifts of triptycene imine H_3_ and pyridine proton H_4_, and an upfield shift of pyridine proton H_5_. The bulky anion tetraphenylborate interacted weakly with **1** at its peripheral openings, as evidenced by upfield shifts of the pyridine H_5_ and imine H_7_ proton signals (Figures S29 and S30).

**Figure 4:**
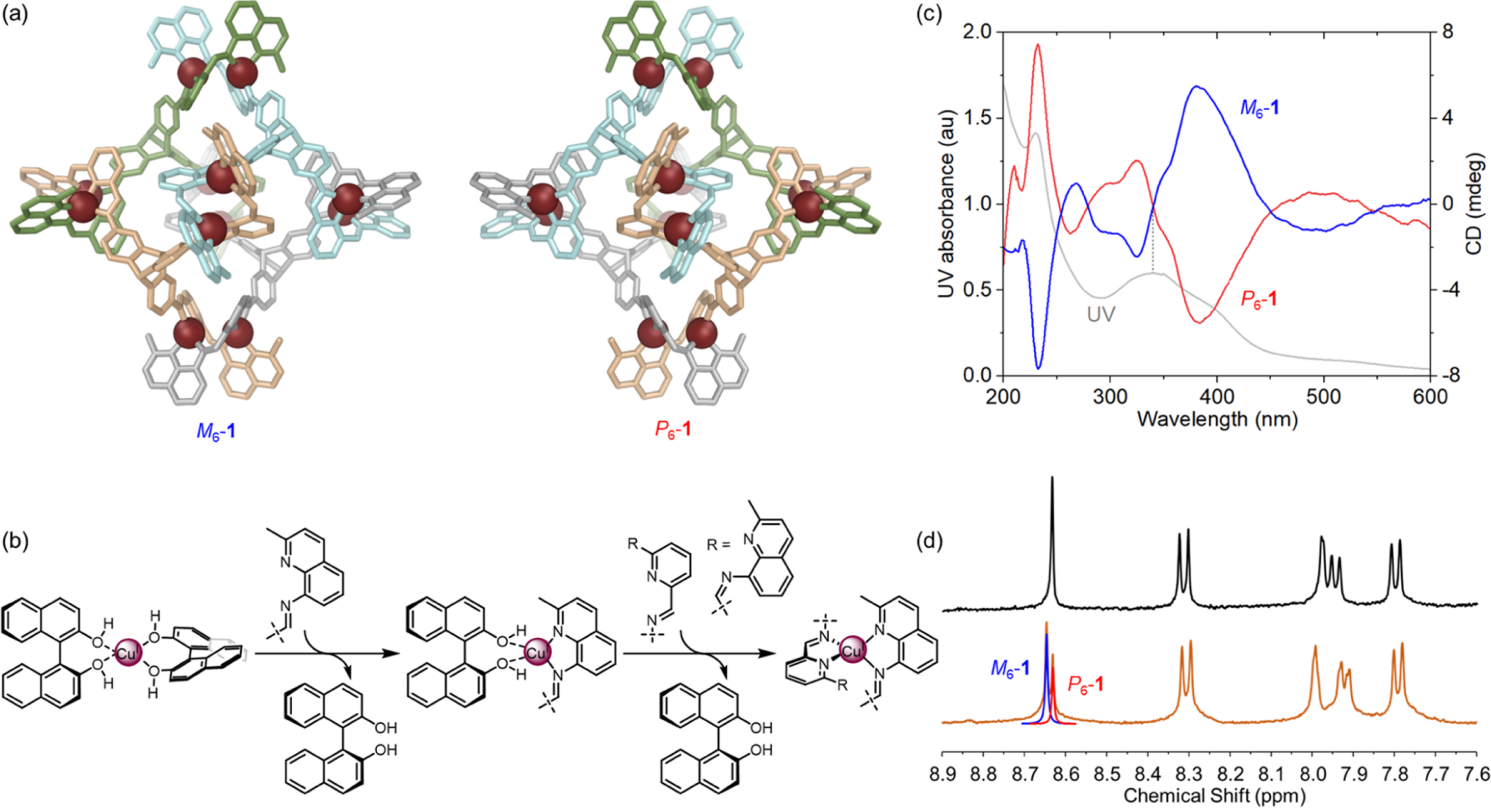
(a) Enantiomers of **1**. Ligands are individually colored to show the helicity. (b) Proposed mechanism of stereoinduction during the formation of the Cu^I^ stereocenters of **1**. (c) UV–Vis and CD spectra of *M*_6_- and *P*_6_-biased **1** in acetonitrile. (d) ^1^H NMR spectra (400 MHz, 298 K, CD_3_CN) of *M*_6_-biased **1** before (top, black) and after (bottom, orange) the addition of 2 equiv of *Δ*-TRISPHAT.

Cage **1** is formed as a racemic mixture, where the corners of each cage consist of either six *M*-helicates or six *P*-helicates (denoted as *M*_6_-**1** and *P*_6_-**1**, respectively, Figure 4a), as reflected in a featureless circular dichroism (CD) spectrum. We envisaged that **1** might be formed stereoselectively through the addition of a chiral auxiliary.^73^ Screening prospective auxiliaries revealed that the presence of enantiopure 1,1′-bi-2-naphthol (BINOL) during the self-assembly of **1** led to stereocontrol (Scheme S3). After removal of the BINOL by washing with ethyl acetate and diethyl ether, cage **1** displayed clear CD Cotton effects from 350 to 450 nm, consistent with chiral induction during its synthesis. Specifically, the use of (*S*)-BINOL (50 equiv) led to a higher population of *P*_6_-**1**, as reflected in a negative Cotton effect at the MLCT band around 400 nm,^69^ whereas (*R*)-BINOL (50 equiv) favored the formation of *M*_6_-**1**, with a mirror-image CD spectrum (Figure 4c). Although BINOL-derived ligands have been employed as building blocks in chiral cages,^74,75^ the use of BINOL as a chiral additive has been less explored.^76^

The enantiomeric excess (*ee*) of **1** prepared in the presence of BINOL was determined by deconvolution and integration of the ^1^H NMR spectra containing tetrabutylammonium *Δ*-tris(tetrachlorocatecholato)phosphate (*Δ*-TRISPHAT) as a chiral discrimination agent.^77−79^ The imine signal H_13_, originally a singlet, split in two upon the addition of 2 equiv of *Δ*-TRISPHAT (Figure 4d). NMR integration indicated an *ee* of 29% was obtained following the use of 100 equiv of BINOL. The intensity of the CD signals did not decrease after the sample was heated to 80 °C for 15 days, indicating a strong chiral memory effect owing to the cooperative locking together of the structure by 48 N → Cu^I^ coordinative bonds (Figure S33).^80^

The effects of BINOL upon the stereoinduction of **1** were further probed by a series of self-assembly experiments (Figures S33–S40). Three parallel reactions were performed, each containing different amounts of (*R*)-BINOL. The intensity of the CD signal at 385 nm was found to correlate positively with the amount of (*R*)-BINOL added. We fitted these data using 1:1 and 1:2 binding models (Figure S36). A 1:2 binding mode provided a better fit and gave a maximum CD intensity at 385 nm of 10.6 mdeg. The *ee* value obtained from NMR integration was also plotted against the amount of (*R*)-BINOL added, and the relationship was also analyzed using the same 1:2 model (Figure S39). The CD intensity at 385 nm was found to correlate linearly with the NMR-determined *ee* of **1** (Figure S40).

The mechanism of chiral induction was probed through a series of control experiments. A racemic sample of **1** was mixed with 50 equiv of (*R*)-BINOL in acetonitrile and heated to 343 K over 36 h. The ratio of *M*_6_-**1** to *P*_6_-**1**, measured by NMR integration, did not change (Figure S41). Heating (*R*)-BINOL with *P*_6_-**1**, or (*S*)-BINOL with *M*_6_-**1**, did not result in a reversal or decrease of the corresponding CD signal, indicating that helical handedness was imprinted by BINOL during the formation of the cage and that the chiral memory effect was impervious to BINOL thereafter. Moreover, the treatment of **1** with BINOL did not cause any NMR chemical shift changes, indicating that host–guest interactions did not occur between **1** and BINOL (Figure S42).

We thus infer that (*S*)- or (*R*)-BINOL coordinates to Cu^I^ initially, yielding a chiral intermediate that undergoes further ligand exchange to stereoselectively produce the helicate corners of the chiral cage framework (Figure 4b). Evidence of complexation between BINOL and Cu^I^ was observed by ^1^H NMR (Figure S43). The nonlinear relationship between CD intensity and *ee* plotted against BINOL concentration (Figures S33–S38) also supports the formation of a Cu^I^(BINOL)_2_ complex, as noted above. Temperature-dependent *ee* and CD results (Figures S44 and S45) provide further support for our putative mechanism, as the weakly bound intermediate BINOL-Cu^I^ complexes would decomplex at higher temperatures, favoring cage formation pathways that do not implicate BINOL.

Stereocontrol during the synthesis of **1** was attempted through the addition of other chiral compounds that contain O, N, or P donor groups, and which may thus serve as bidentate or monodentate ligands to Cu^I^ (Figure S46). In all cases, either no stereoselectivity was observed or **1** was not formed (Figures S47 and S48). We thus conclude that BINOL operates in a “sweet spot”, not binding so strongly to Cu^I^ that cage formation is inhibited but binding strongly enough to bias helical handedness.

**Figure 5:**
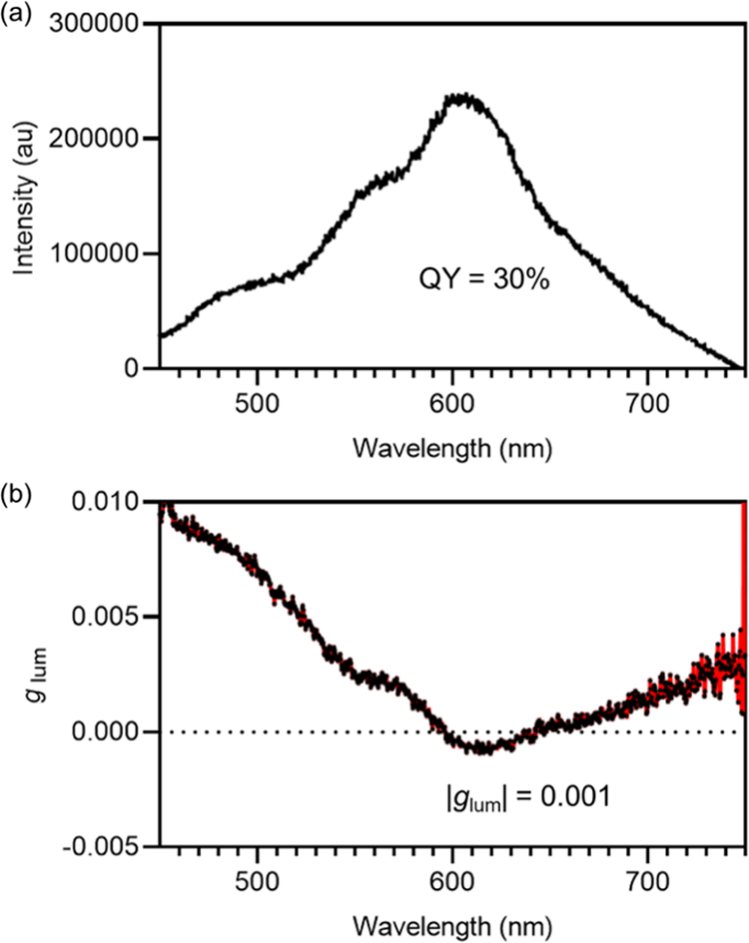
(a) PL spectrum of **1** in acetonitrile, at an excitation wavelength of 400 nm and a concentration of 10^–5^ M. (b) Determination of the |*g*_lum_| of **1** in acetonitrile.

We hypothesized that stereoselectively prepared **1** would emit circularly polarized light, as the MLCT states of copper(I) diimine complexes are known to be emissive.^81^ A solution of **1** in acetonitrile was observed to luminesce following laser excitation at 400 nm, with an absolute quantum yield (QY) of 30% (Figure 5a). The steady-state photoluminescence (PL) spectrum revealed a broad emission from 400 to 750 nm, centered at 610 nm. Time-resolved PL analysis confirmed that the emission faded rapidly over the nanosecond time scale, precluding the presence of triplet-state-involved radiative decay. CPL measurements revealed that the helicity in **1** exhibited a dissymmetry factor (|*g*_lum_|) of 0.001 (Figure 5b). While the lack of f electrons endows the cage with only a moderate |*g*_lum_|, our results demonstrate the embedding of dicopper(I) helicate moieties within a cage framework to be effective for the fabrication of novel structures for CPL.

## Conclusions

The formation of cage **1** thus validates a strategy of integrative self-sorting during the subcomponent self-assembly of complex structures, as each equivalent of symmetrical dialdehyde **B** must react with one **A** and one **C** to form the helicate corners. This strategy may also be extended to the preparation of larger cages that combine multiple subcomponents, for example, incorporating tetratopic or pentatopic amines with nonplanar cores. The six helicate corner structures also cooperatively increase the stability of **1**, as evidenced by its stereochemical memory effect, which should benefit its potential applications in other solvents, potentially including water, for stereoselective binding. Moreover, the method of stereoselective synthesis through the use of BINOL demonstrates a new mode of transferring stereochemical information from the environment during cage synthesis, potentially avoiding tedious covalent modifications of ligands with chiral substituents.

## Data Availability

Computational structures and outputs are available at 10.5281/zenodo.10372347.

## Supporting Information Available

Experimental procedures, NMR spectra, photophysical results, and computational details (PDF)
