# Supplementary material for: Stereocontrolled Self-Assembly of a Helicate-Bridged CuI12L4 Cage That Emits Circularly Polarized Light
Source: J Am Chem Soc. 2024 Jan 22;146(4):2379–86. doi: 10.1021/jacs.3c11321 (PMC10835658; doi:10.1021/jacs.3c11321)
Supplement: Supplementary file 1 — ja3c11321_si_001.pdf [file ja3c11321_si_001.pdf]

Stereocontrolled self-assembly of a helicate-bridged $Cu^{I}_{12}L_4$ cage that emits circularly-polarized light

Huangtianzhi Zhu, Luca Pesce, Rituparno Chowdhury, Weichao Xue, Kai Wu, Tanya K. Ronson, Richard H. Friend, Giovanni M. Pavan,* Jonathan R. Nitschke**

Table of Contents

1. Materials and methods……………………………………………………………………………S2

2. Synthesis and characterization of **1**...………………………………………………………………S3

3. Self-assemblies with different subcomponents and Cu^{I} salts……………………………………S10

4. Host–guest studies of **1**……………...……………...............................……………………….S17

5. Stereochemical studies of **1**…………………………........................……………………………S24

6. Photophysical studies of **1**……….………................................……………………………………S36

7. Computational optimization of the cage structure…………..............……………………S38

8. References……………………...……………………………………………..……………………......S43

1. Materials and methods

All starting materials were purchased from commercial sources and used as supplied. 2,7,14-trinitrotriptycene was prepared according to a published procedure.[1] (*S*)- and (*R*)-BINOL were purchased from Sigma Aldrich and used as received. Solvents were used as supplied. A CEM Discover microwave reactor was used for the subcomponent self-assembly of **1**.

NMR spectra were recorded using 400 MHz Avance III HD Smart Probe (routine ^{1}H NMR, DOSY) and DCH 500 MHz dual cryoprobe (high-resolution ^{13}C and 2D experiments) NMR spectrometers. Chemical shifts (δ) for ^{1}H NMR spectra are reported in parts per million (ppm) and are reported relative to the solvent residual peak. Coupling constants (J) were reported in Hz to 1 decimal place. ^{1}H DOSY NMR experiments were conducted on a Bruker 400 MHz Avance III HD Smart Probe spectrometer. Maximum gradient strength was 5.35 G/cm A. The standard Bruker pulse program, ledbpgp2s,3 employing a stimulated echo and longitudinal eddy-current delay (LED) using bipolar gradient pulses for diffusion using 2 spoil gradients, was utilized. A gradient ramp of 5% to 90% was used. High-resolution electrospray ionisation mass spectra were recorded on a Waters Synapt G2-Si instrument.

2. Synthesis and characterization of **1**

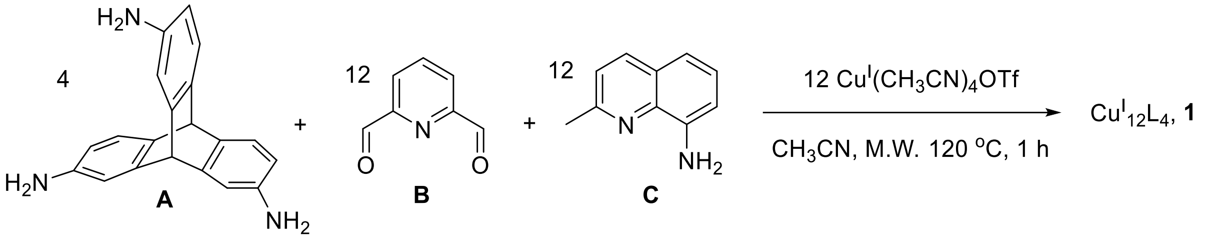

Scheme S1. Synthesis of **1**.

A (1.00 mg, 4 equiv, 3.34 μmol), **B** (1.35 mg, 12 equiv, 10.0 μmol), **C** (1.58 mg, 12 equiv, 10.0 μmol) tetrakis(acetonitrile)copper(I) triflate (3.76 mg, 12 equiv, 10.0 μmol) and 0.50 mL of acetonitrile were added into a small vial that was sealed in a glove box. The vial was kept at 393 K in a microwave reactor for 1h, affording a dark brown suspension. The solvent was reduced by nitrogen flow, followed by the addition of ethyl acetate. The precipitate was collected by centrifugation and washed with ethyl acetate and diethyl ether. After drying in vacuum, **1** was obtained as a black solid in approximately quantitative yield.

The same cage can also be prepared by heating the reaction mixture at 323 K overnight in an oil bath. Microwave is not required to prepare this cage.

^{1}H NMR (500 MHz, 298 K, CD_3CN) δ 9.78 (s, 12H), 8.65 (s, 12H), 8.32 (d, J = 8.5 Hz, 12H), 7.99 (d, J = 2.3 Hz, 12H), 7.94 (dd, J = 7.8, 1.0 Hz, 12H), 7.80 (dd, J = 8.4, 1.2 Hz, 12H), 7.44 (d, J = 8.5 Hz, 12H), 7.40 (d, J = 7.9 Hz, 12H), 7.24 (dd, J = 7.8, 1.1 Hz, 12H), 7.12 (t, J = 7.9 Hz, 12H), 7.00 (dd, J = 7.8, 2.3 Hz, 12H), 6.58 (dd, J = 7.6, 1.2 Hz, 12H), 6.19 (s, 4H), 5.86 (t, J = 7.7 Hz, 12H), 5.80 (s, 4H), 1.71 (s, 36H).

^{13}C NMR (126 MHz, 298 K, CD_3OD) δ 160.1, 157.6, 157.5, 149.3, 149.1, 146.4, 145.8, 143.0, 142.5, 140.7, 137.6, 136.4, 130.6, 130.3, 128.5, 126.9, 126.8, 125.1, 124.9, 124.2, 122.5, 120.0, 116.2, 53.6, 51.7, 24.0.

High-resolution ESI-MS: m/z = 827.5053 [**1** + $(OTf)_5]^{7+}$, 990.0831 [**1** + $(OTf)_6]^{6+}$, 1218.0914 [**1** + $(OTf)_7]^{5+}$, 1560.1028 [**1** + $(OTf)_8]^{4+}$, 2129.4583 [**1** + $(OTf)_9]^{3+}$.

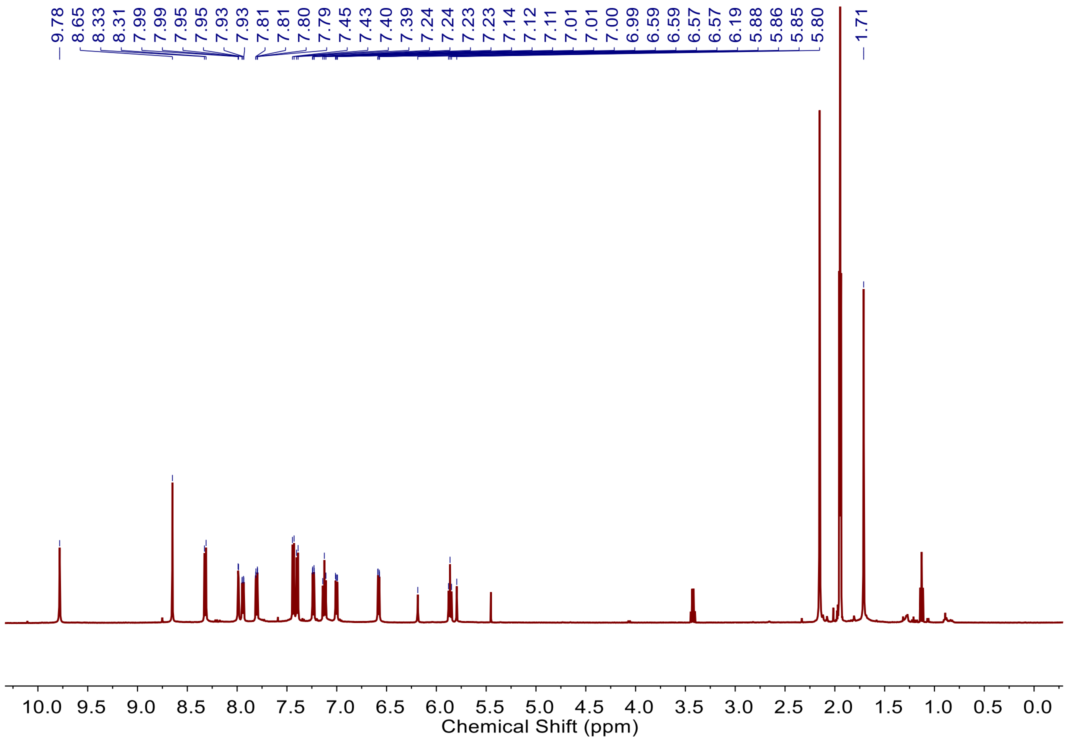

Figure S1. ^{1}H NMR spectrum (500 MHz, 298 K, CD_3CN) of **1**.

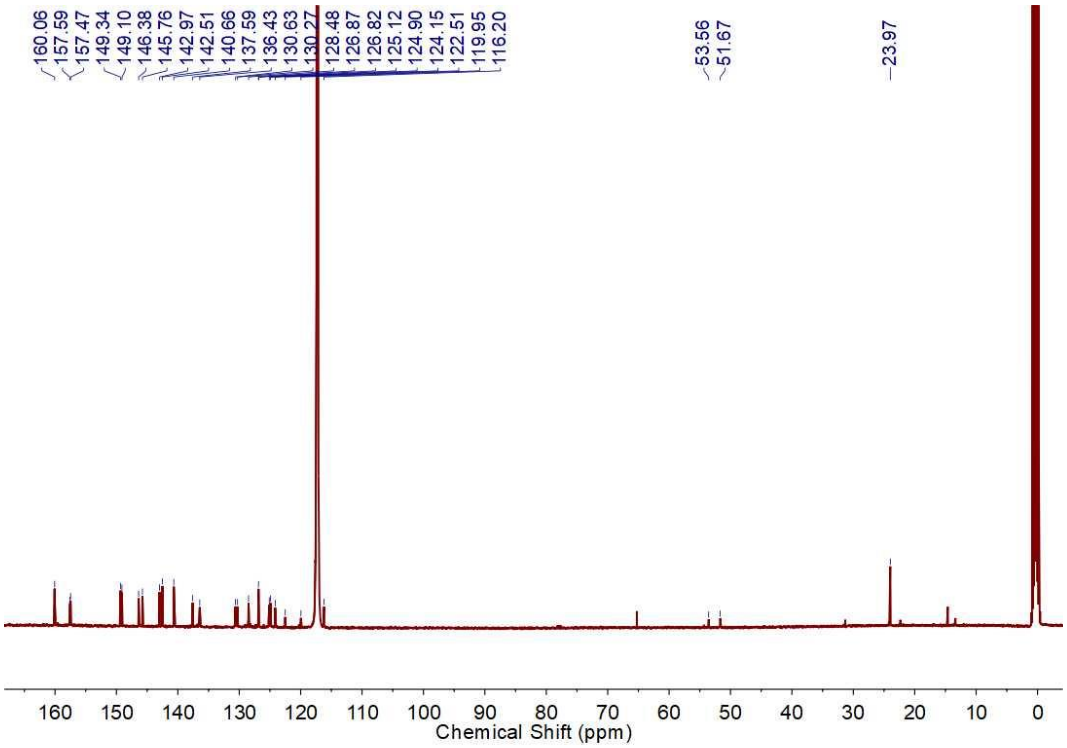

Figure S2. ^{13}C NMR spectrum (126 MHz, 298 K, CD_3CN) of **1**.

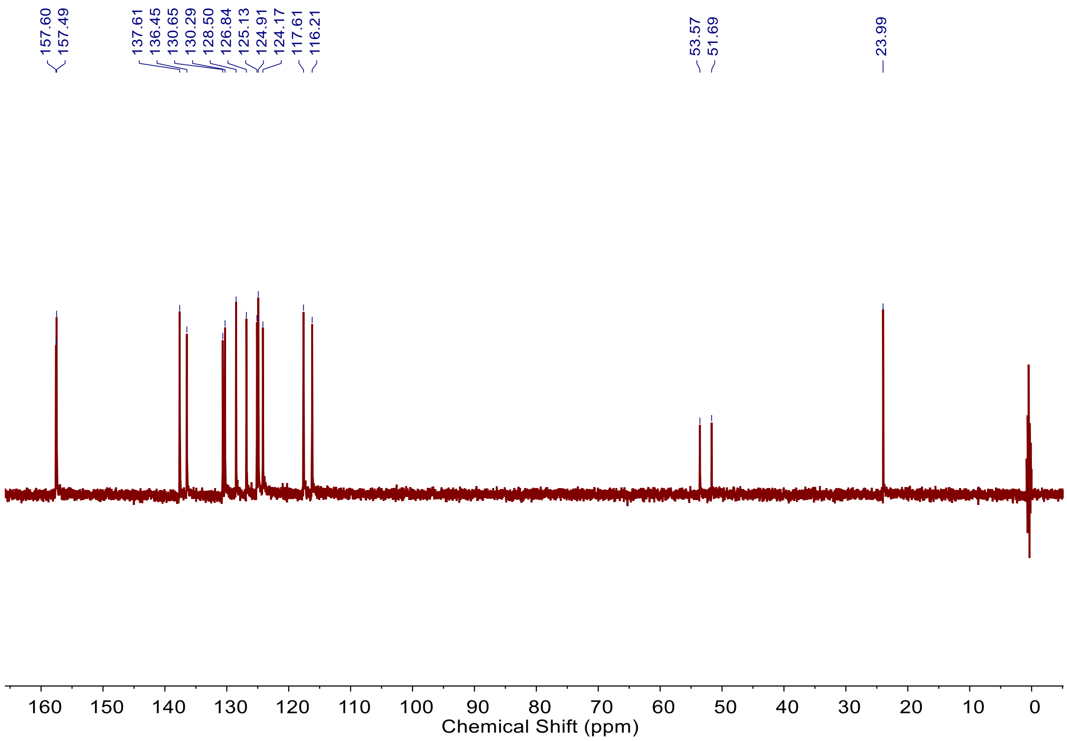

Figure S3. ^{13}C dept NMR spectrum (126 MHz, 298 K, CD_3CN) of **1**.

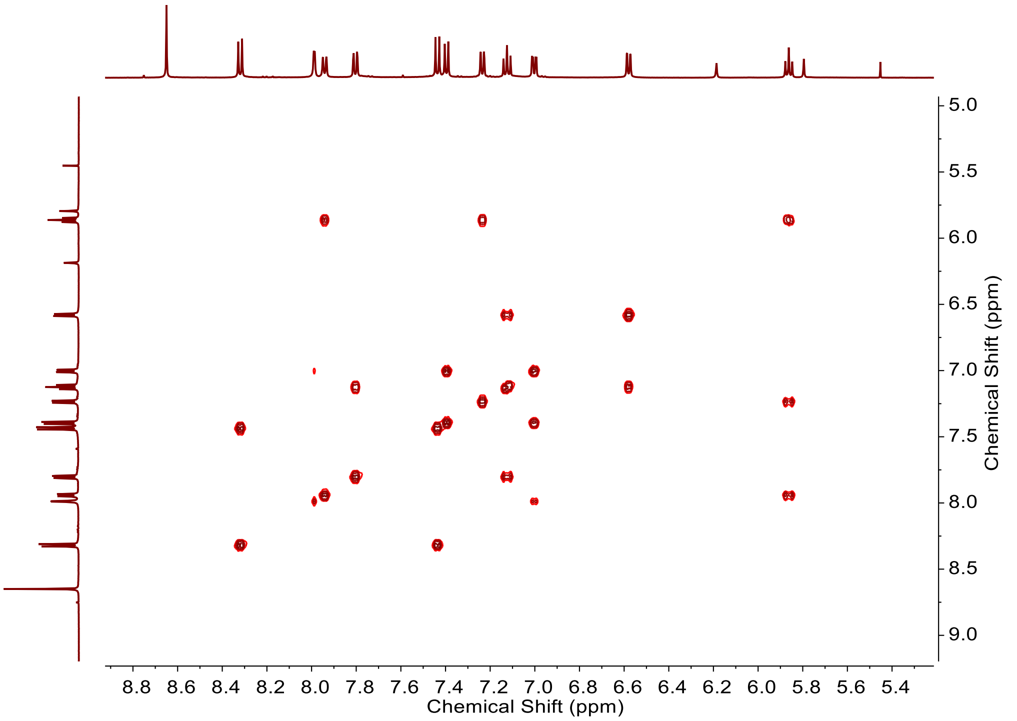

Figure S4. ^{1}H-^{1}H COSY spectrum (500 MHz, 298 K, CD_3CN) of **1**.

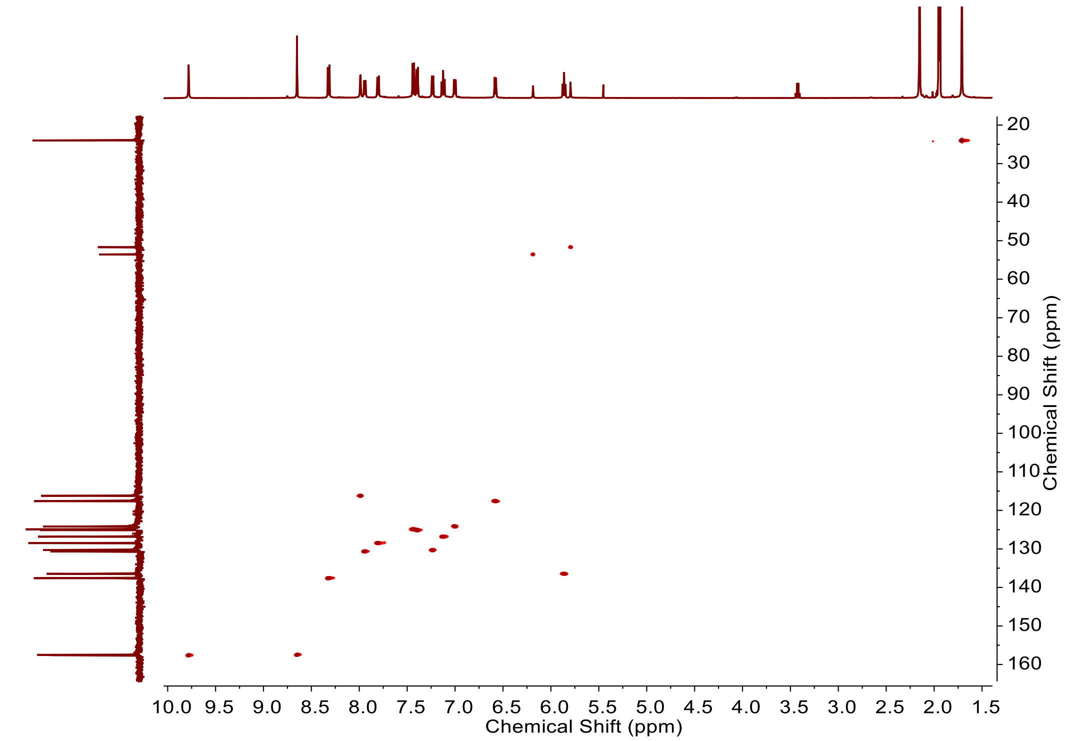

Figure S5. ^{1}H-^{13}C HSQC spectrum (500 MHz, 298 K, CD_3CN) of **1**.

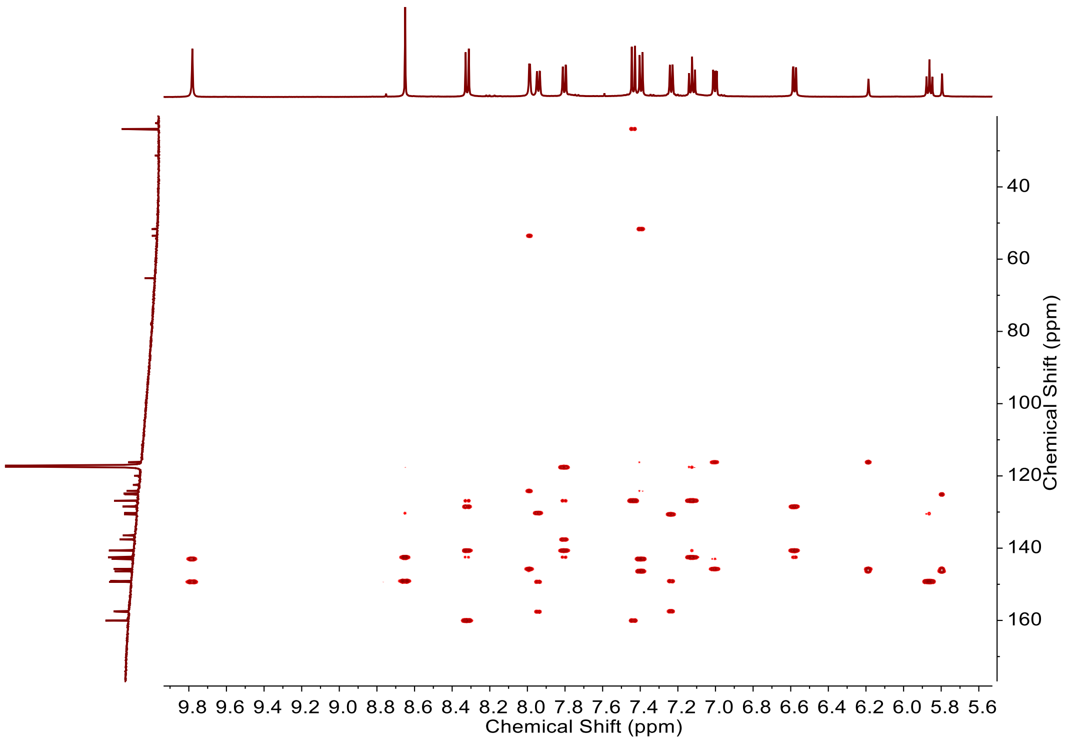

Figure S6. ^{1}H-^{13}C HMBC spectrum (500 MHz, 298 K, CD_3CN) of **1**.

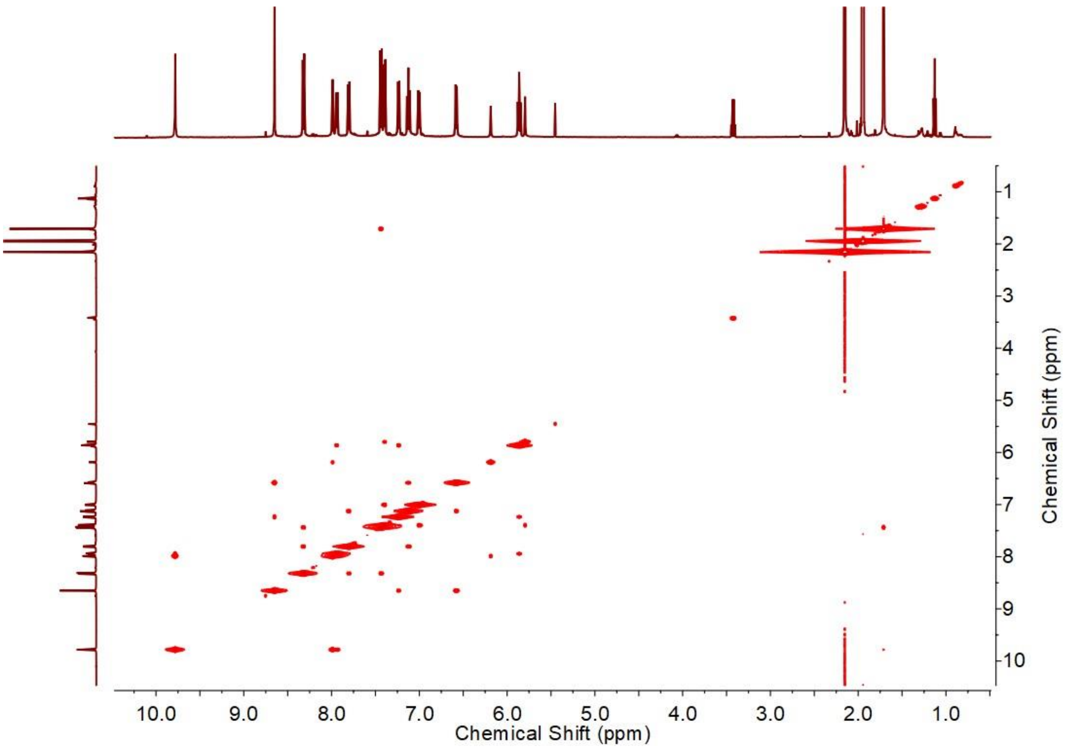

Figure S7. ^1H-^1H NOESY spectrum (500 MHz, 298 K, CD_3CN) of **1**.

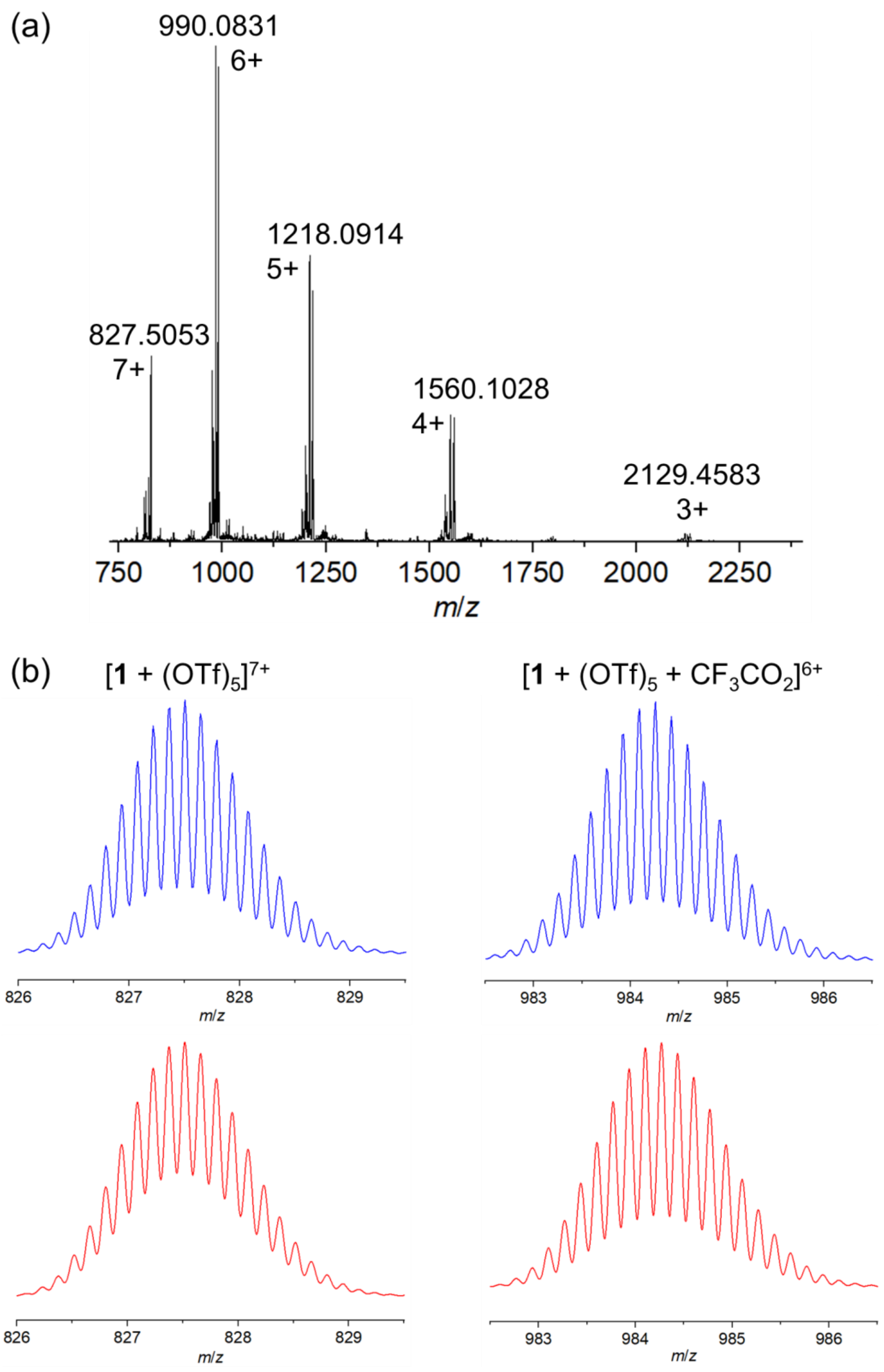

Figure S8. High-resolution ESI-mass spectrum of **1**: (a) Full spectrum and (b) selected cations (7+ and 6+, from left to right) with experimental (blue) and calculated (red) isotopic distributions. Replacement of OTf^{-} with $CF_3CO_2^{-}$ was observed within the mass spectrometer, giving a distribution of $[\mathbf{1} + (OTf)_n + (CF_3CO_2)_m]^{12-n-m}$.

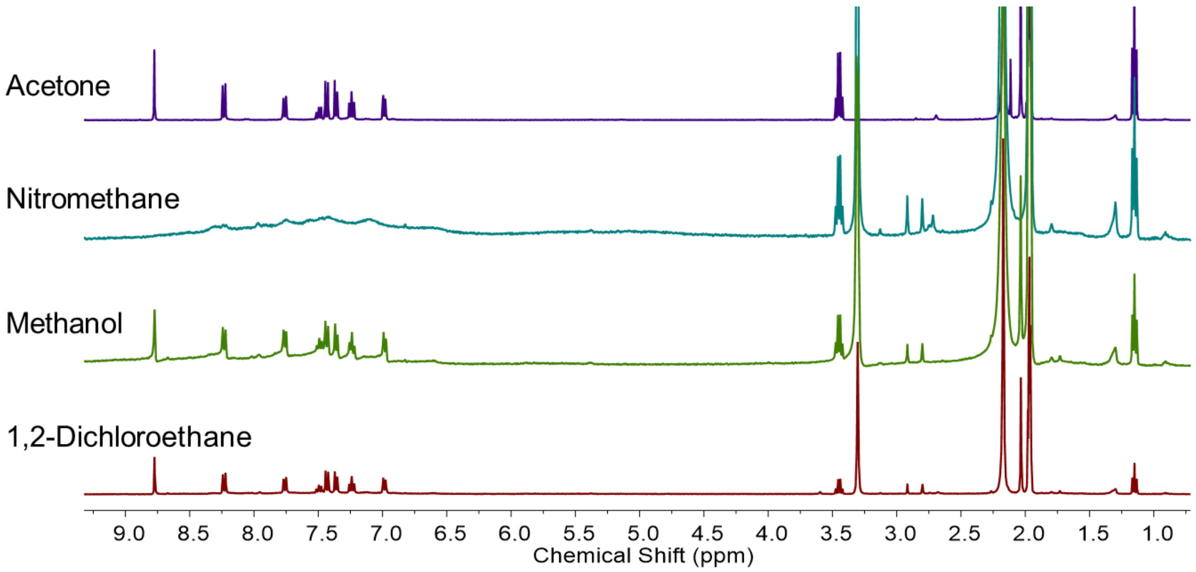

Figure S9. ^{1}H NMR spectra (400 MHz, 298 K, CD_3CN) of assembly reactions carried out in different solvents. Only the dicopper(I) helicate was obtained in acetone, methanol and 1,2-dichloroethane. No trace of **1** was observed. We infer that as acetonitrile is a good ligand for copper(I), the self-assembly process in acetonitrile is more reversible, enabling the annealing processes that result in self-sorting into the cage structure instead of random oligomers. The reaction in other solvents results in kinetically-trapped insoluble polymers incorporating triaminotriptycene. Thus we only observed the soluble dicopper(I) helicate in solution.

3. Self-assemblies with different subcomponents and Cu^{I} salts

The same structure can be prepared using 4-bromo-2,6-diformylpyridine and 8-aminoquinaline as subcomponents together with $Cu^{I}(CH_3CN)_4X$ ($X = OTf^-$, BF_4^-, or ClO_4^-), following the procedure described above. All these reactions afforded the identical cage framework, as evidenced by NMR spectroscopy. These small counteranions are not encapsulated in the cavity as no chemical shift differences are observed in the ^{1}H NMR spectra of cages **S1**–**S3**.

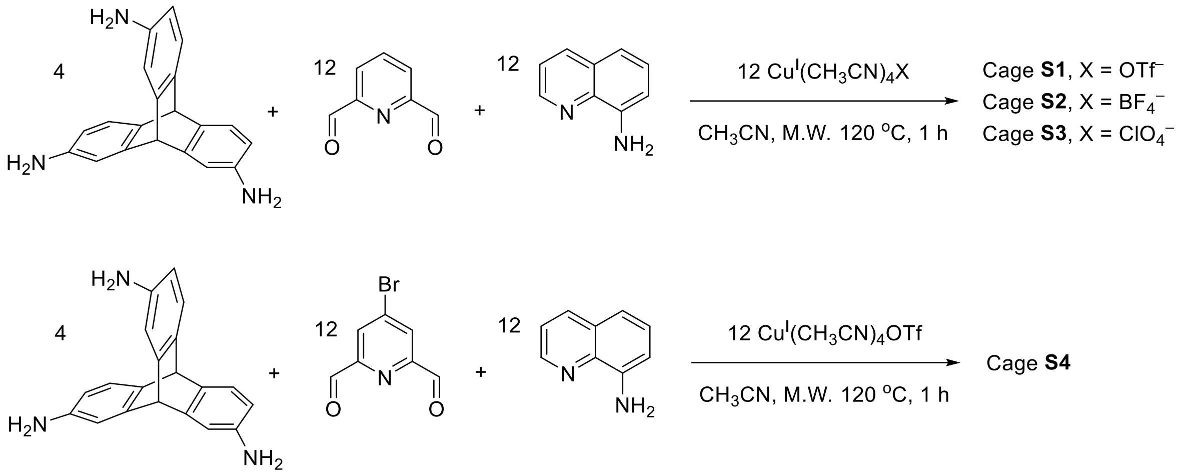

Scheme S2. Synthesis of Cage **S1**–**S4**.

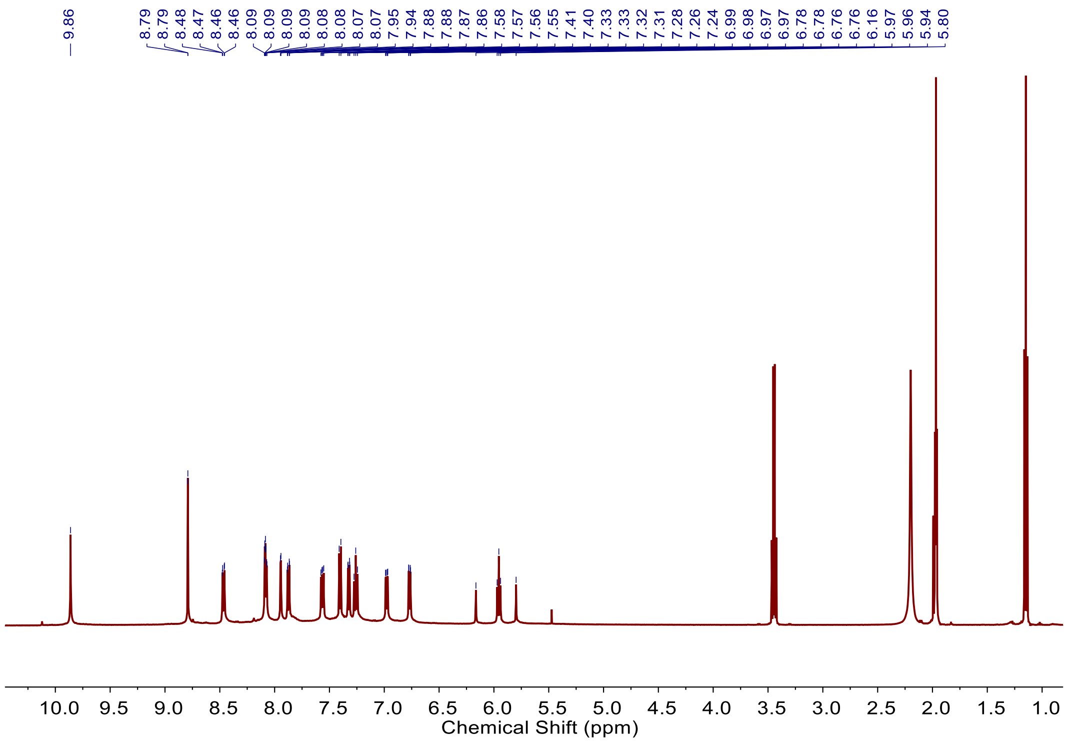

Figure S10. ^{1}H NMR spectrum (400 MHz, 298 K, CD_3CN) of Cage **S1**.

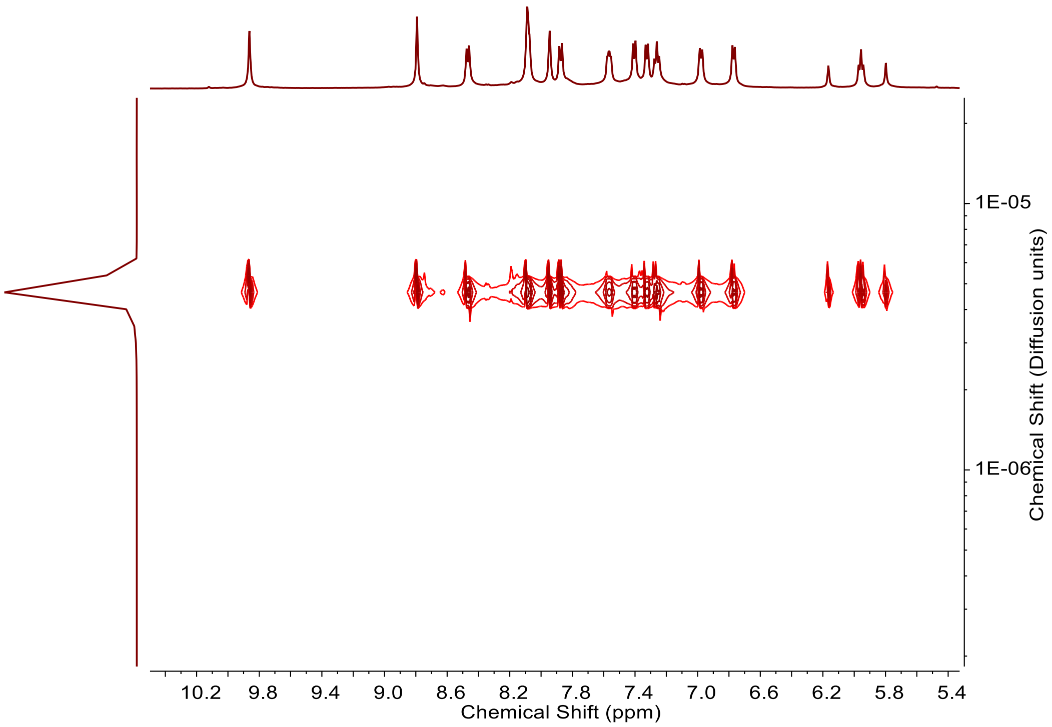

Figure S11. ^{1}H DOSY spectrum (400 MHz, 298 K, CD_3CN) of Cage **S1**.

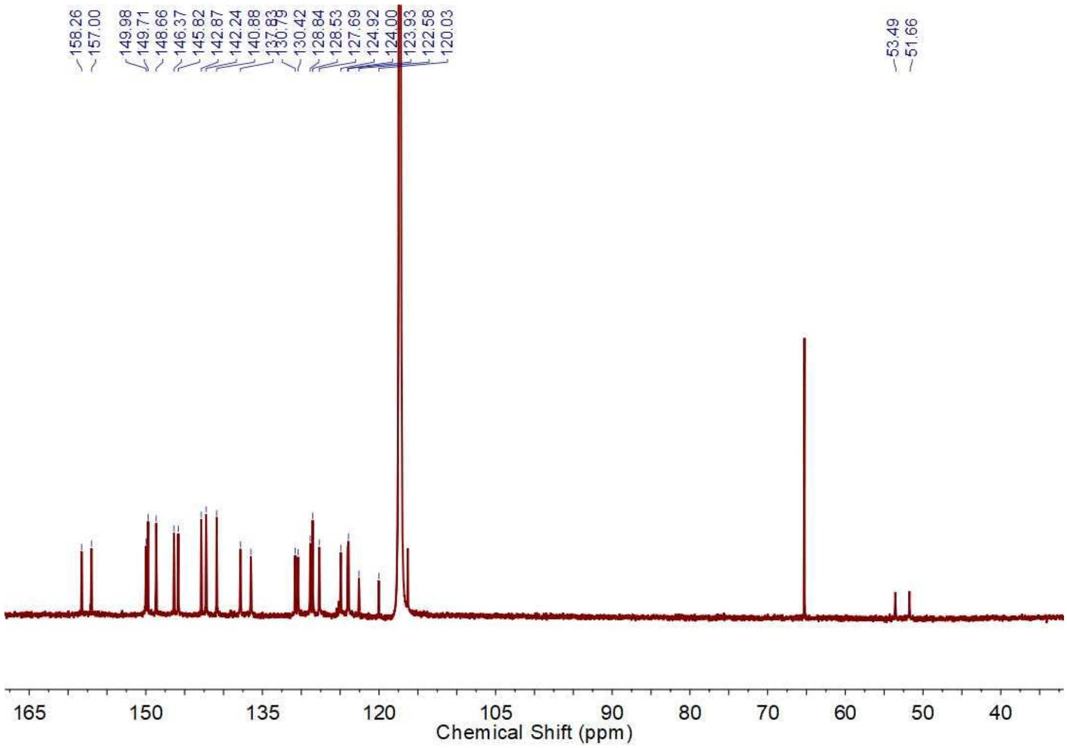

Figure S12. ^{13}C NMR spectrum (100 MHz, 298 K, CD_3CN) of Cage **S1**.

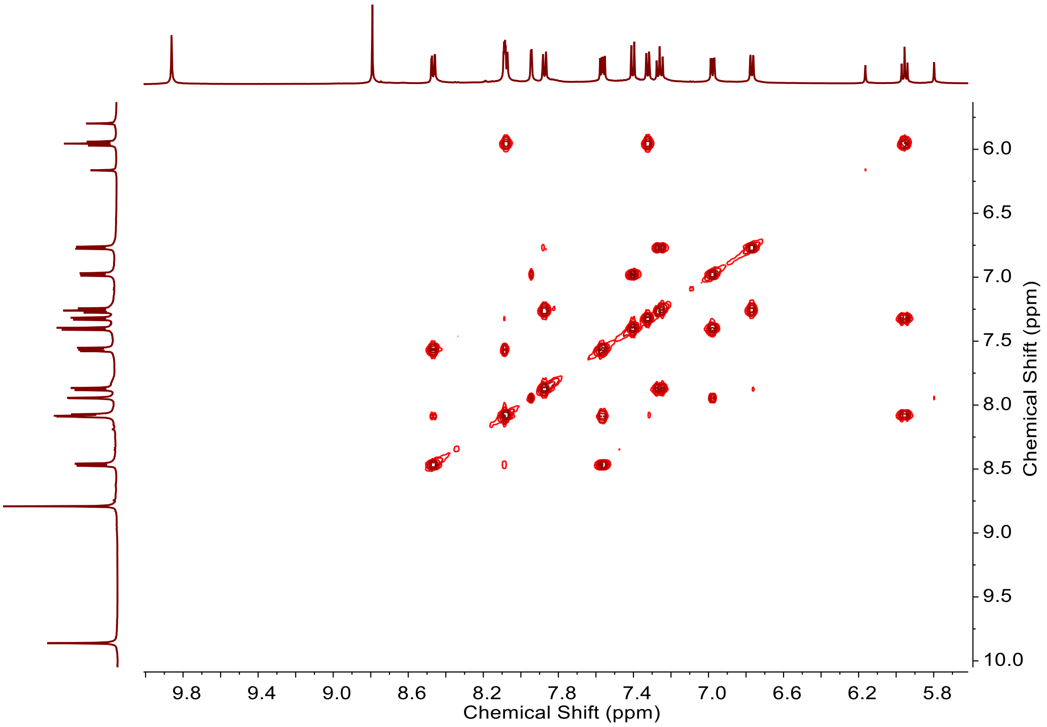

Figure S13. ^{1}H-^{1}H COSY spectrum (400 MHz, 298 K, CD_3CN) of Cage **S1**.

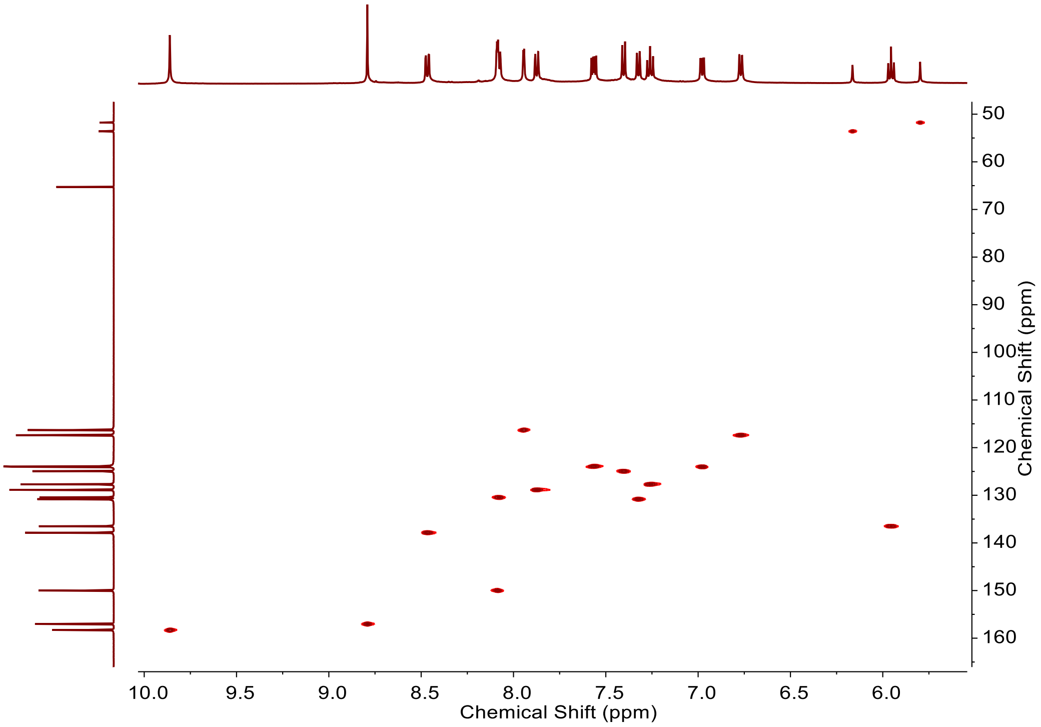

Figure S14. ^{1}H-^{13}C HSQC spectrum (400 MHz, 298 K, CD_3CN) of Cage **S1**.

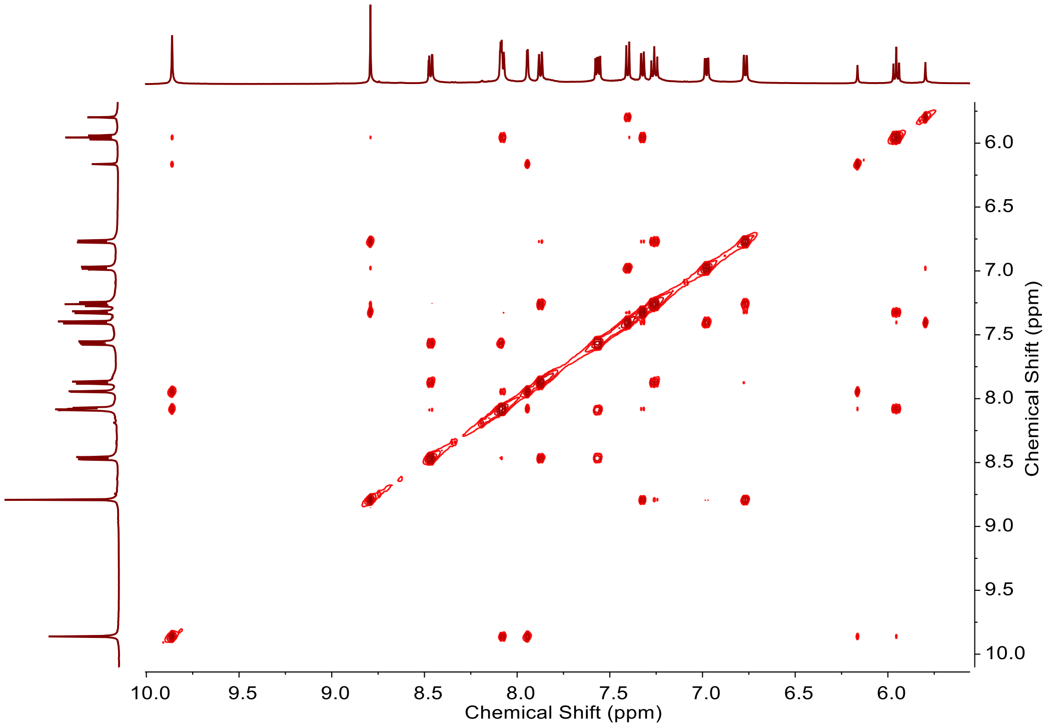

Figure S15. ^{1}H-^{1}H NOESY spectrum (400 MHz, 298 K, CD_3CN) of Cage **S1**.

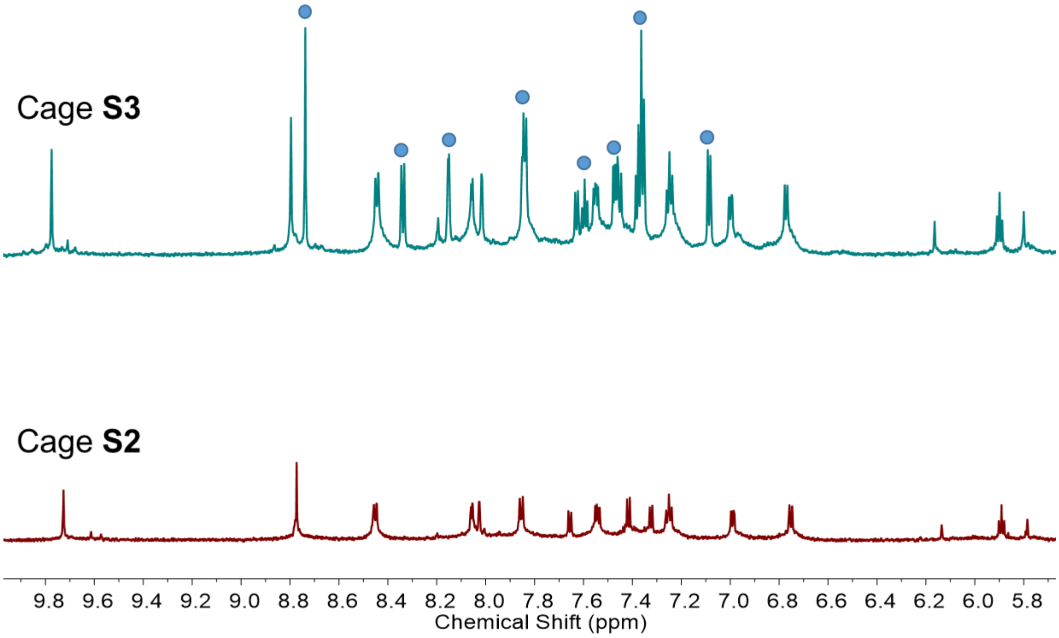

Figure S16. ^{1}H NMR spectra (400 MHz, 298 K, CD_3CN) of Cage **S2** and Cage **S3**. The peaks from the dicopper(I) helicate formed from excess diformylpyridine and aminoquinoline are marked by blue circles.

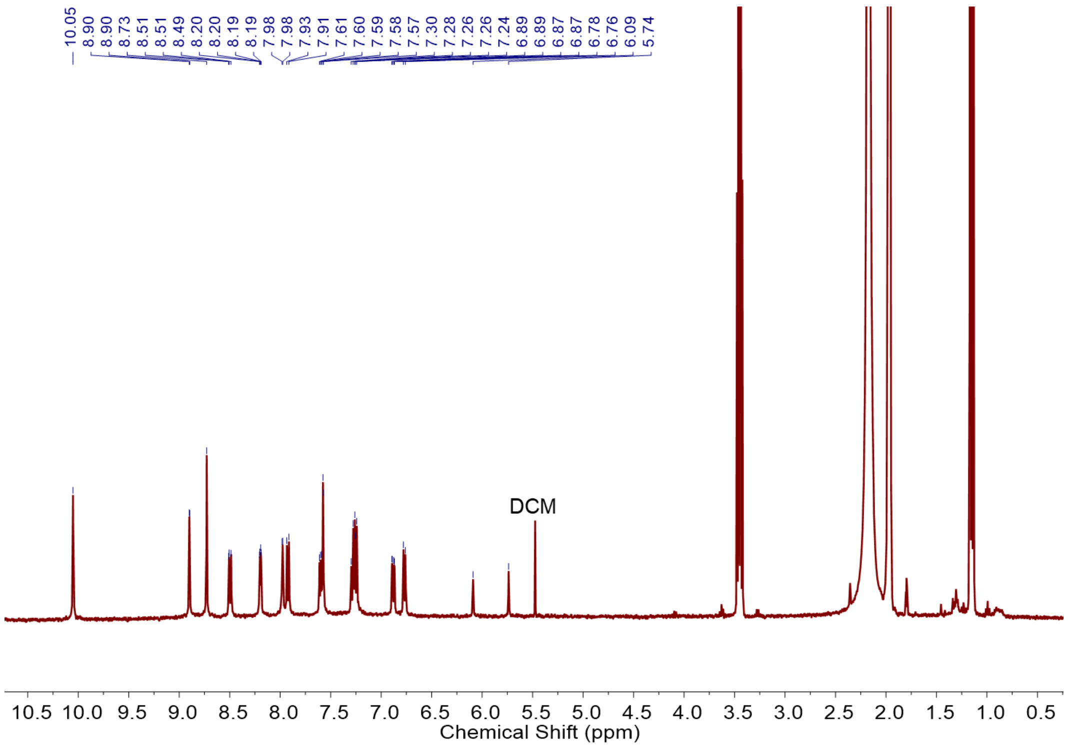

Figure S17. ^{1}H NMR spectra (400 MHz, 298 K, CD_3CN) of Cage **S4**. The absence of the triplet between the two singlets of the alkyl bridges is consistent with the 4-position of pyridine being substituted by bromide.

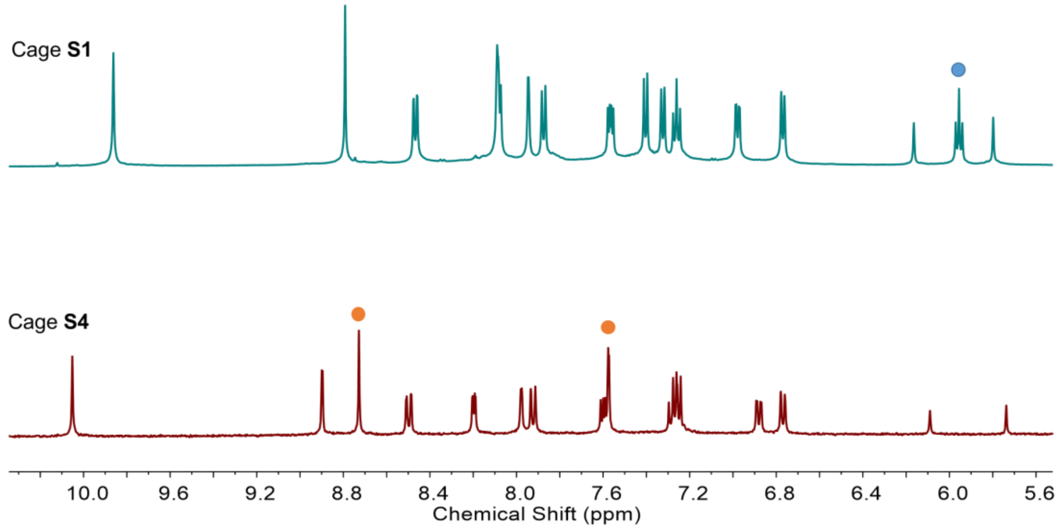

Figure S18. Aromatic region of the ^{1}H NMR spectra (400 MHz, 298 K, CD_3CN) of Cage **S1** and **S4**. The absence of the triplet (marked with a blue circle) between the two singlets of the alkyl bridges is consistent with the the 4-position of pyridine being substituted by bromide. Orange circles represent two singlets on the substituted pyridine ring.

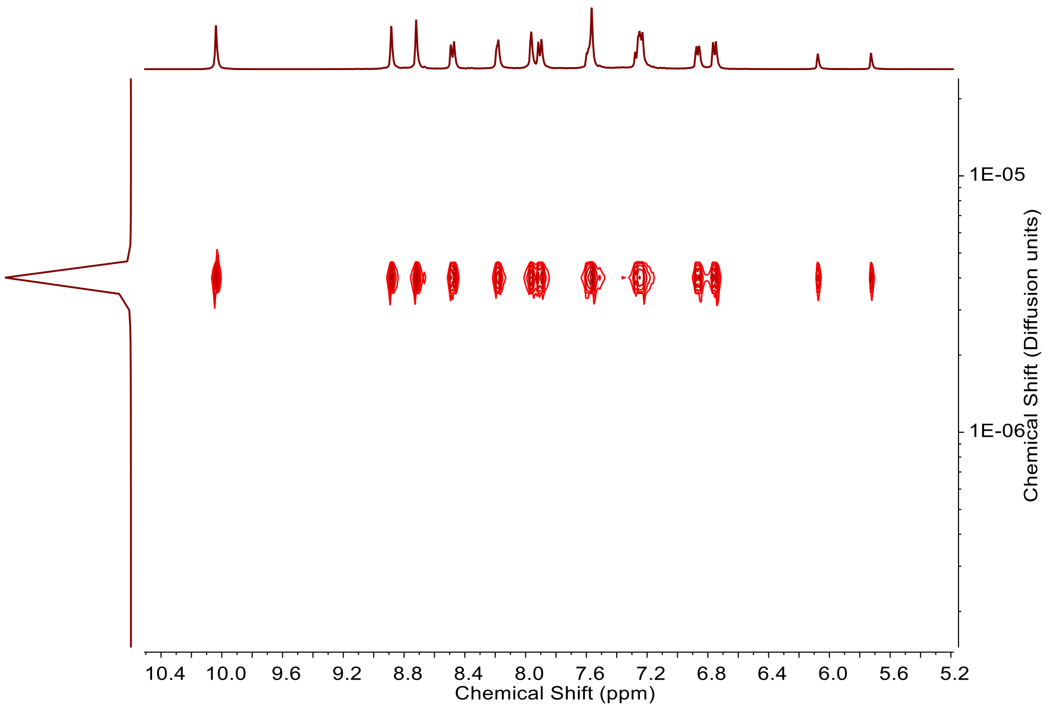

Figure S19. ^{1}H DOSY spectrum (400 MHz, 298 K, CD_3CN) of Cage **S4**.

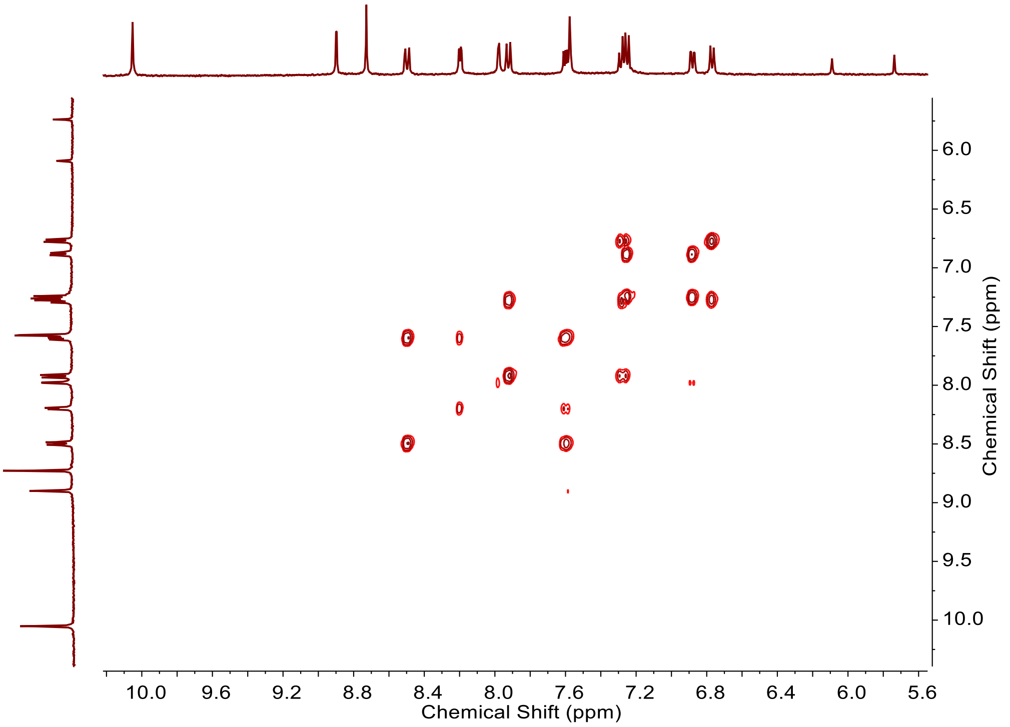

Figure S20. ^{1}H-^{1}H COSY spectrum (400 MHz, 298 K, CD_3CN) of Cage **S4**.

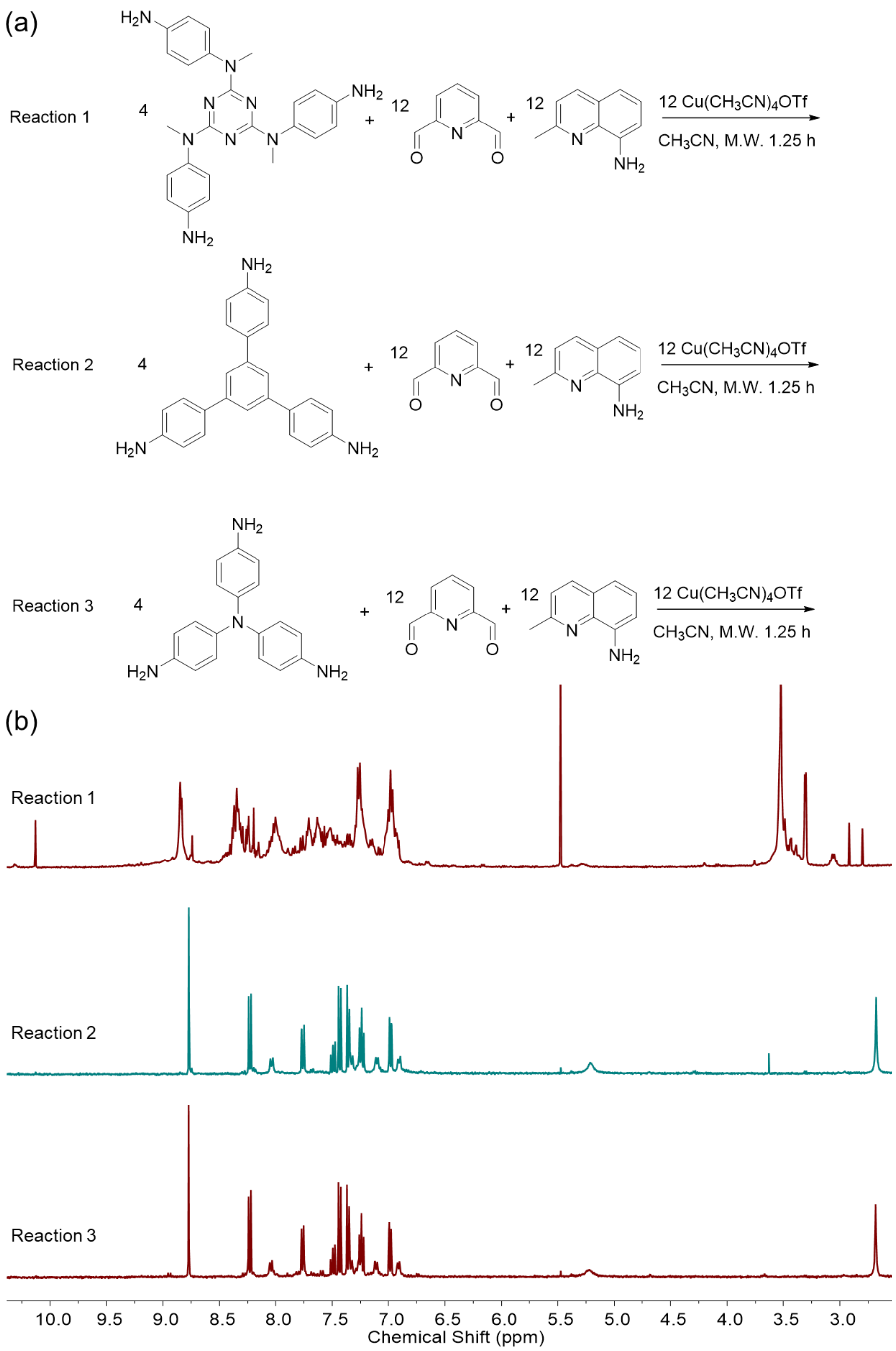

Figure S21. (a) Self-assemblies with planar tritopic subcomponents. (b) ^{1}H NMR spectra (400 MHz, 298 K, CD_3CN) of the self-assemblies. No discrete structure was observed in the self-assembly reaction 1. Only simple dicopper(I) helicates were obtained from reactions 2 and 3, where rigid tritopic ligands formed insoluble polymers that precipitated out. The result suggested the curvature of triptycene was required for the preparation of **1**.

4. Host–guest studies of **1**

The cavity size of **1** was determined to be 344 Å^3 by MoloVol (probe size 1.8 Å).[2] However large windows and the lack of aromatic walls impede the binding of neutral guests. NMR titrations indicated sodium 1-hexylsulfonate, potassium perfluoro-1-hexanesulfonate and tetrabutylammonium tetraphenylborate were complexed by **1**, respectively.

1 was prepared in 0.5 mL CD_3CN with a concentration of 0.0005 M; sodium 1-hexylsulfonate, potassium perfluoro-1-hexanesulfonate and tetrabutylammonium tetraphenylborate stock solutions were prepared in CD_3CN with a concentration of 0.05 M. Known volumes of the guest solution were added into the host solution in an NMR tube. ^{1}H NMR spectra were collected after shaking and sonicating the host-guest mixture for 1-5 minutes.

Bindfit was used to determine the binding constant.[3] Higher binding stoichiometry was excluded due to the small cavity size and repulsion between anions.

The Hill equation was also employed to determine the apparent binding constant and the Hill coefficient.

$$\log(i/(1-i)) = n\log[G] + n\log K_a$$

where i is the fraction of host bound by the guest which is determined by observed chemical shifts ($\Delta\delta$) against the maximum chemical shift during titrations ($\Delta\delta_{max}$), [G] is the guest concentration, n is the Hill coefficient describing cooperativity, and K_a is the apparent association constant. The coefficient $n > 1$ indicates positively cooperative binding, $n = 1$ indicates non-cooperative binding, and $n < 1$ indicates negatively cooperative binding.

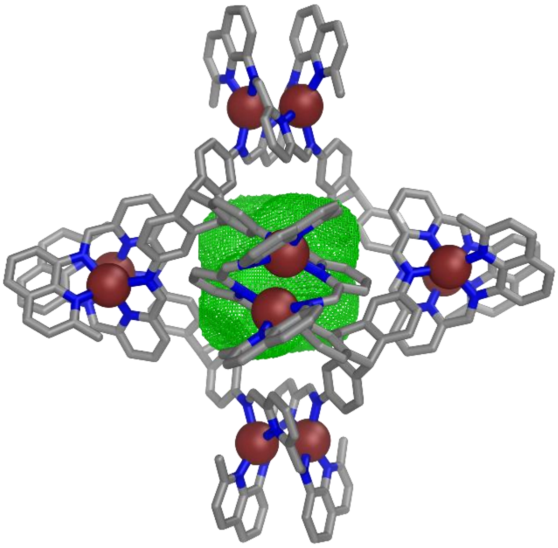

Figure S22. Cavity map (green mesh) of **1**. Carbon atoms are grey, nitrogen atoms are blue, hydrogen atoms are white and copper atoms are ruby.

Titration of 1-hexylsulfonate sodium salt

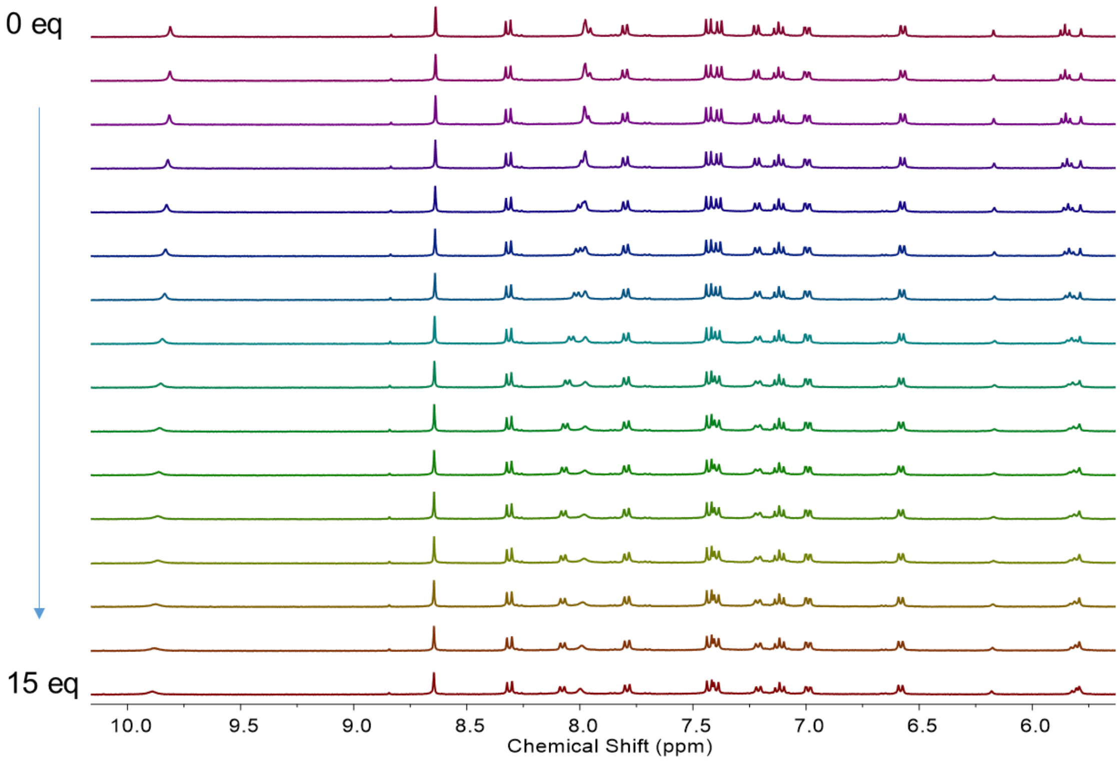

Figure S23. NMR titration of **1** with sodium 1-hexylsulfonate002E

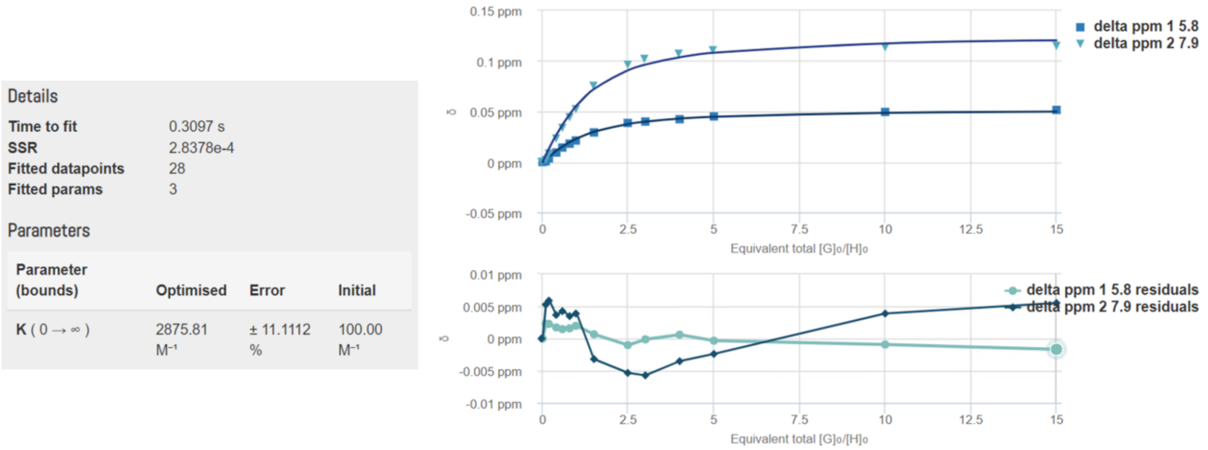

Figure S24. Binding isotherms and residual plots for the binding sodium 1-hexylsulfonate by **1** using BindFit with the 1:1 binding mode, K_a = 2876 M^{-1}, error = ± 11%. The cavity size of the cage and electronic repulsion may exclude the binding of more than one anionic guest.

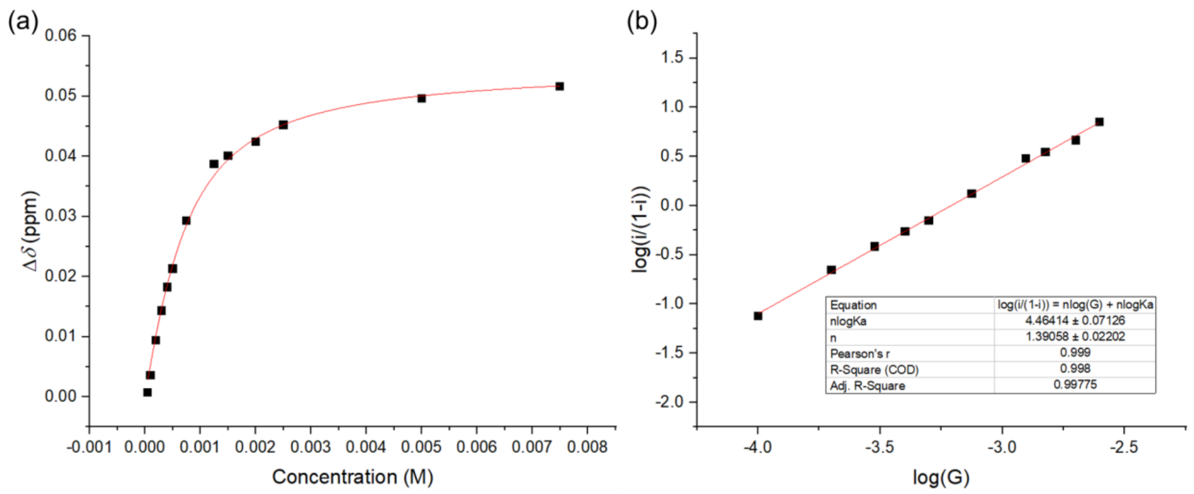

Figure S25. Hill function of the titration of **1** with sodium 1-hexylsulfonate.(a) Titration curve. (b) Hill function fitting. The apparent binding constant was determined to be 1616 M^{-1}. n was determined to be 1.39, which is close to 1.

Titration of potassium perfluoro-1-hexanesulfonate

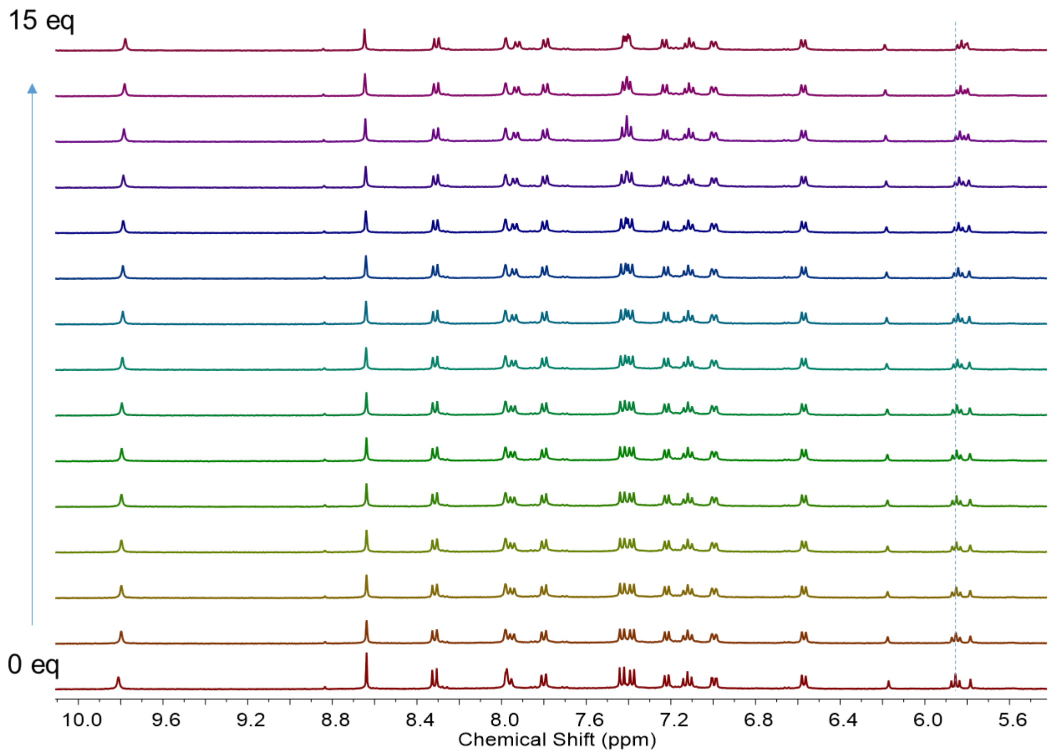

Figure S26. NMR titration (left) of potassium perfluoro-1-hexanesulfonate and the corresponding non-linear curve fitting (right) for the associate constant.

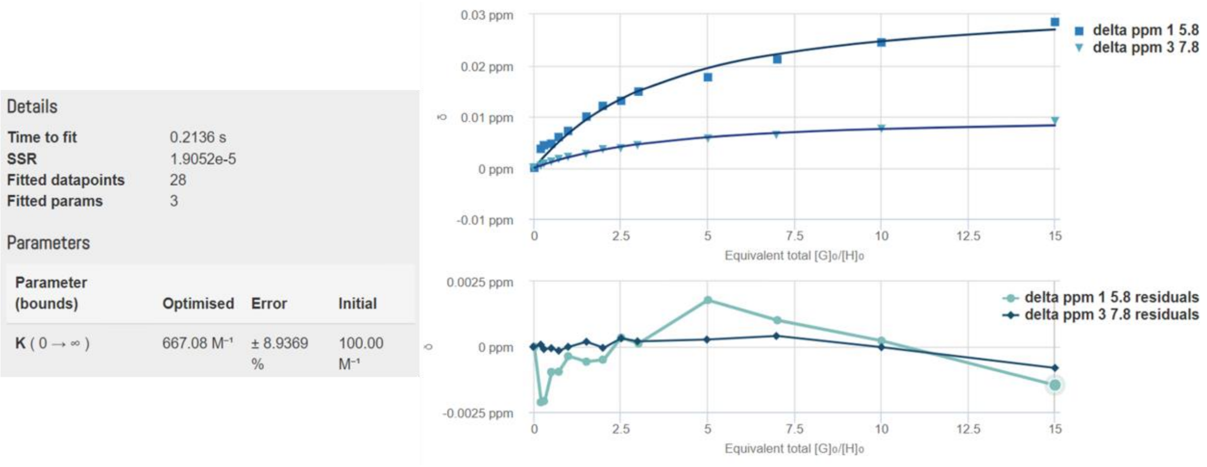

Figure S27. Binding isotherms and residual plots of binding potassium perfluoro-1-hexanesulfonate by **1** using BindFit with the 1:1 binding mode, K_a = 667 M^{-1}, error = ± 9%. The cavity size of the cage and electronic repulsion may exclude the binding of more than one anionic guest.

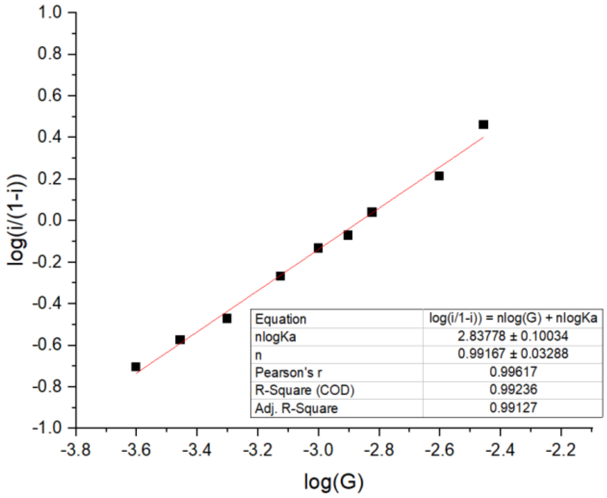

Figuer S28. Hill function of the titration of **1** with potassium perfluoro-1-hexanesulfonate. The apparent binding constant was determined to be 727 M^{-1}, close to the result from Bindfit. *n* was determined to be 0.99, which is close to 1.

Titration of tetrabutylammonium tetraphenylborate

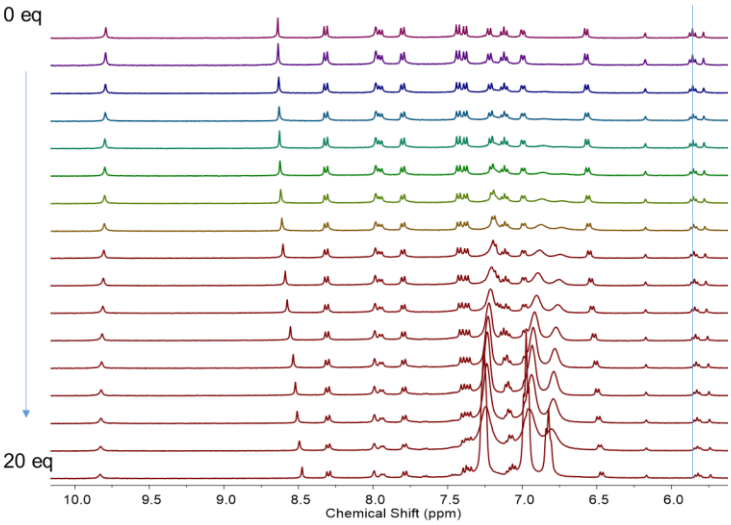

Figure S29. NMR titration (left) of tetrabutylammonium tetraphenylborate and the corresponding non-linear curve fitting (right) for the associate constant. The chemical shift changes occur from the protons that locate on the cage surface, rather than those pointing into the cavity. We therefore infer a peripheral binding mode.

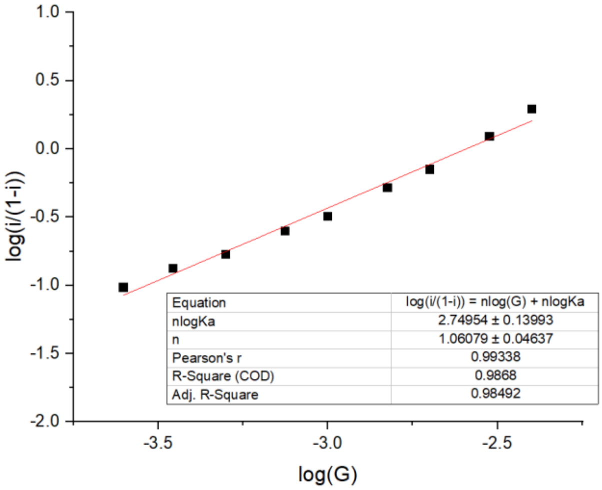

Figuer S30. Hill function of the titration of **1** with tetrabutylammonium tetraphenylborate. The apparent binding constant was determined to be 384 M^{-1}. n was determined to be 1.06, which is close to 1. Note that the cavity size of the cage exclude internal binding of this anion, therefore the stoichiometry of such fast-exchange peripheral binding is hard to be determined. Only Hill function is employed to determine the binding constant.

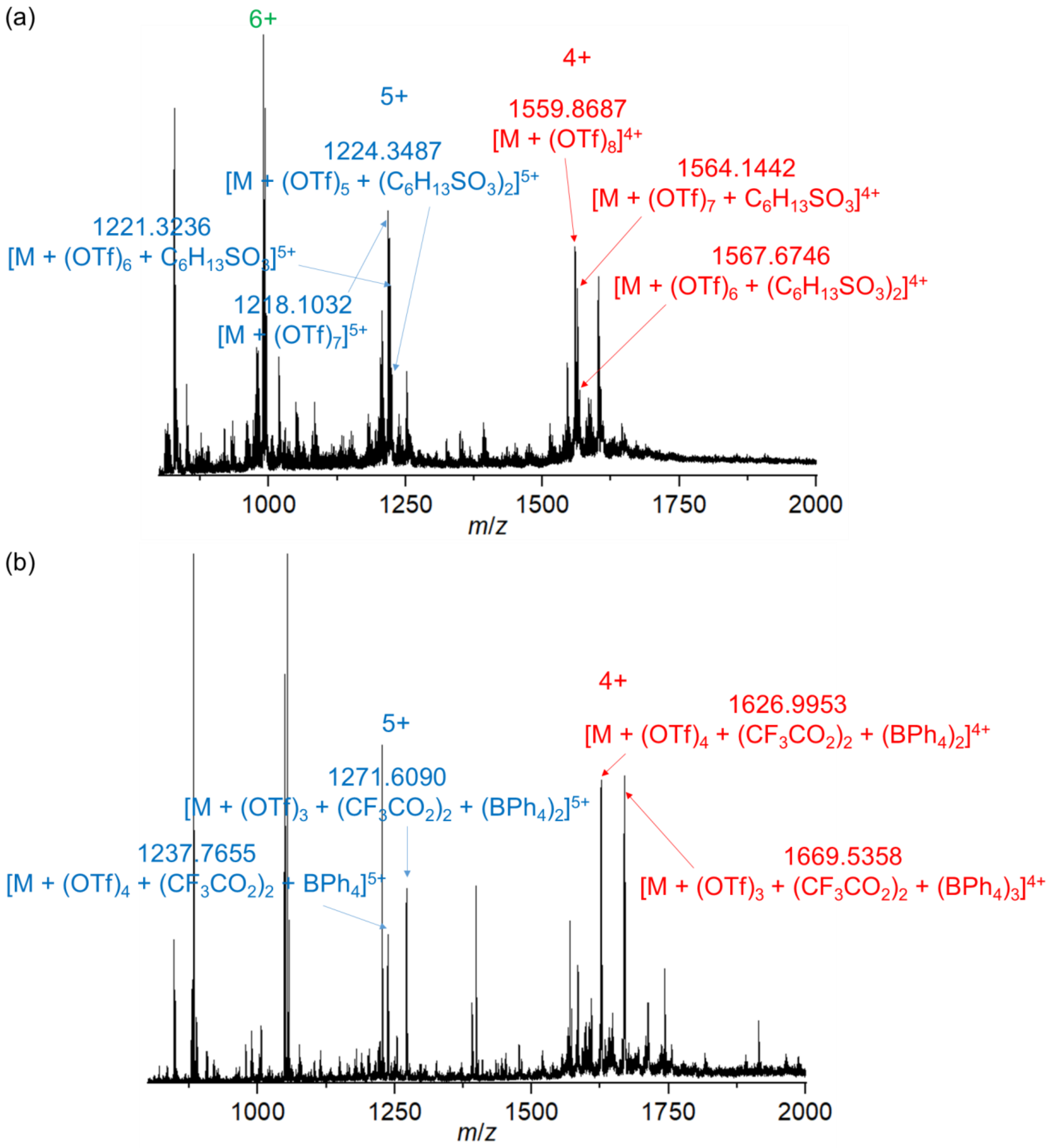

Figure S31. High-resolution ESI-mass spectrum of **1** in the presence of (a) 1-hexylsulfonate sodium salt and (b) tetrabutylammonium tetraphenylborate.

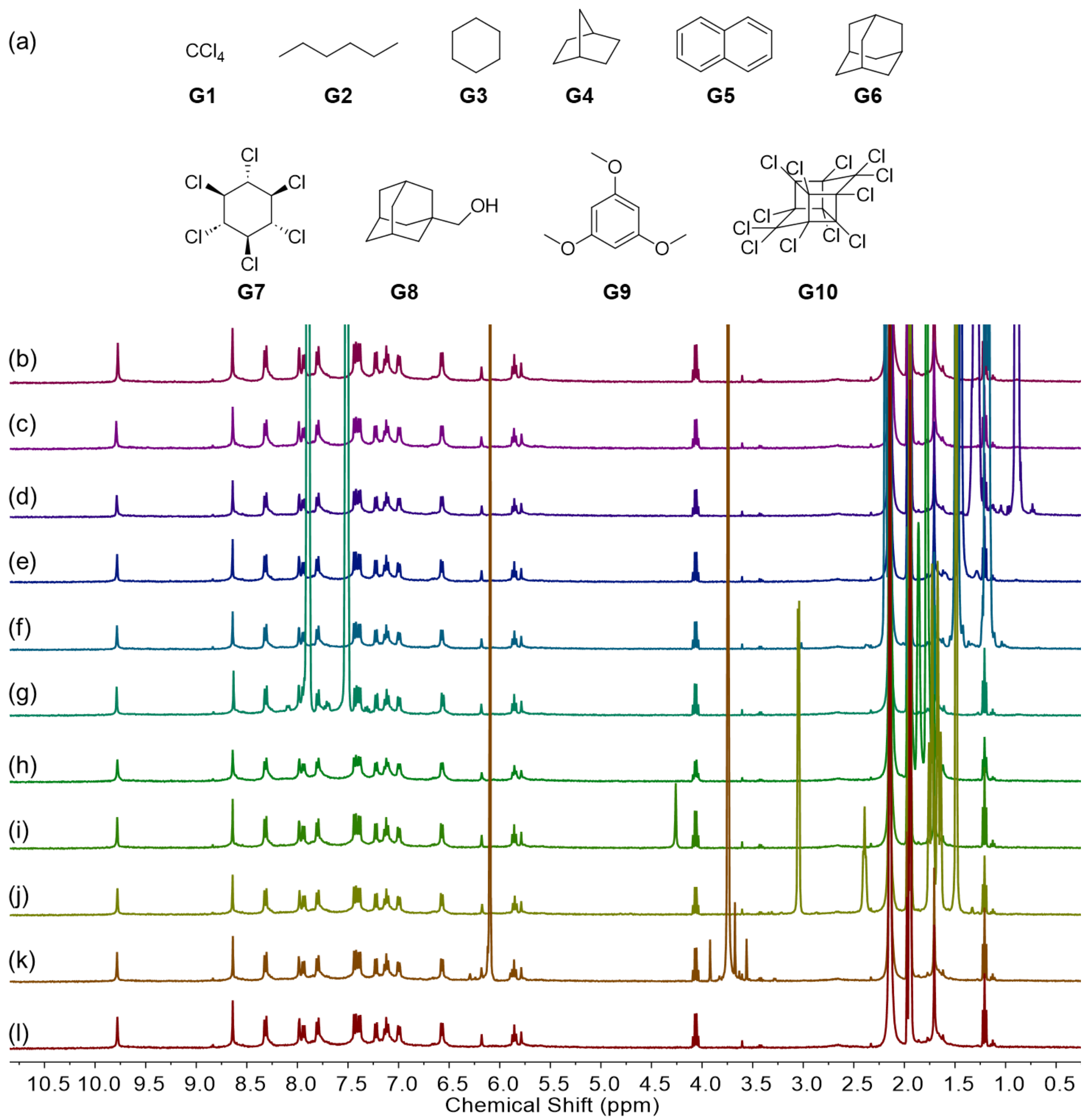

Figure S32. (a) Non-binding neutral guests tested for cage **1**. 1H NMR (400MHz, 298 K, CD_3CN) of (b) **1**, (c) **1** + **G1**, (d) **1** + **G2**, (e) **1** + **G3**, (f) **1** + **G4**, (g) **1** + **G5**, (h) **1** + **G6**, (i) **1** + **G7**, (j) **1** + **G8**, (k) **1** + **G9** and (l) **1** + **G10**. The concentration of the host is 1 mM. The guest concentration is 10 mM.

5. Stereochemical studies of **1**

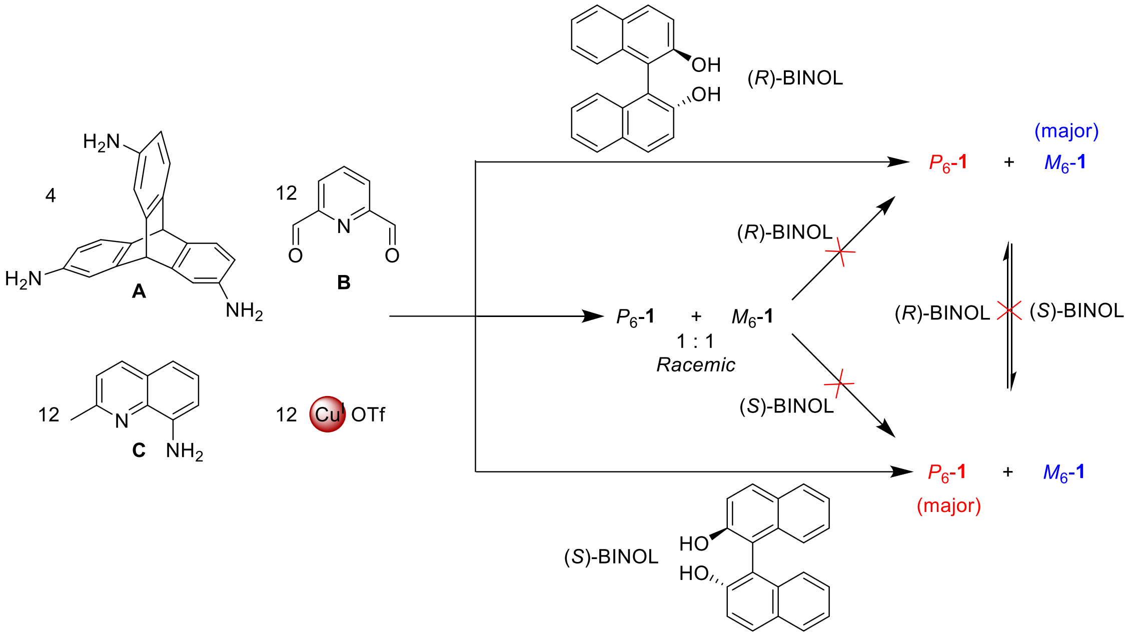

Scheme S3. Stereochemical studies of **1**.

Preparation of P_6- or M_6-biased **1**: Tetrakis(acetonitrile)copper(I) triflate (3.76 mg, 12 equiv, 10.0 μmol), (*S*) or (*R*)-BINOL (various equivalents relative to subcomponent **A**) and 0.50 mL of acetonitrile were added into a small vial that was sealed in the glove box. The solution was stirred for 2 h. **A** (1.00 mg, 4 equiv, 3.34 μmol), **B** (1.35 mg, 12 equiv, 10.0 μmol) and **C** (1.58 mg, 12 equiv, 10.0 μmol) were then added into the solution and the mixture was heated at 323 K in an oil bath overnight, affording a dark brown suspension. For chiral induction with BINOL, oil bath heating at lower temperatures gave better stereocontrol. We infer that at lower temperatures, the chiral intermediate $Cu^{I}(BINOL)_2$ complex, is more stable, producing a higher *ee*. The solvent was reduced by nitrogen flow, followed by the addition of ethyl acetate. The precipitate was collected by centrifugation and washed with ethyl acetate and diethyl ether to remove BINOL completely, as confirmed by ^{1}H NMR. After drying under vacuum, P_6- or M_6-biased **1** was obtained as a black solid in approximately quantitative yield. The maximum amount of BINOL tested was 100 equiv. Higher amounts of BINOL caused precipitation and decreased the yield of the cage.

Identical NMR spectra were obtained as those of racemic **1**. CD spectra were recorded by diluting the solution of **1** to 1 × 10^{-5} M (calibrated by UV absorbance) in acetonitrile. Pure acetonitrile was used to determine the baselines for UV-vis and CD spectra. *Δ*-TRISPHAT (2.00 equivalents relative to **1**) was added to NMR samples of P_6- or M_6-biased **1** to enable chiral discrimination. The proton signal of H_7 on the quinaldine imine splits into two peaks. After deconvolution, the areas of the two peaks were calculated to determine the *ee* value of **1**.

The M_6-biased **1** used in the experiments described below was prepared in the presence of (*R*)-BINOL. Equivalents of (*R*)-BINOL used are shown in each caption. (*R*)-BINOL was completely removed prior to any tests.

Chiral memory and studies of racemization

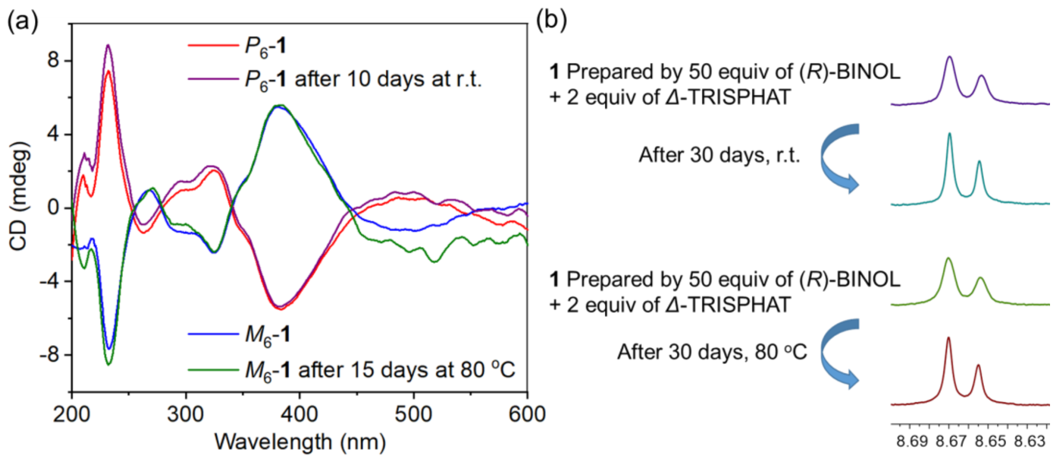

Figure S33. (a) CD spectra of enantioenriched samples of **1**, immediately after synthesis and after 10 days at either room temperature or 80 °C. (b) ^{1}H NMR spectra (400 MHz, 298 K, CD_3CN) of **1** prepared in the presence of 50 equiv of BINOL, containing 2 equiv of *Δ*-TRISPHAT. BINOL was only used in the preparation of **1** and was not present during the 30-day periods noted. The same CD intensities in (a) and integrated peak areas of M_6- and P_6-**1** in (b) indicated the stereochemistry of **1** is stable, with a strong chiral memory effect. Although the *Δ*-TRISPHAT used in the NMR tests might have a stereochemical effect that stabilizes one enantiomer, the consistency between CD and NMR results confirmed that the chiral memory effect was not due to the presence of *Δ*-TRISPHAT. Note that different time periods and temperatures were used to test the stereochemical stability.

Relationships between CD, ee and equivalents of BINOL

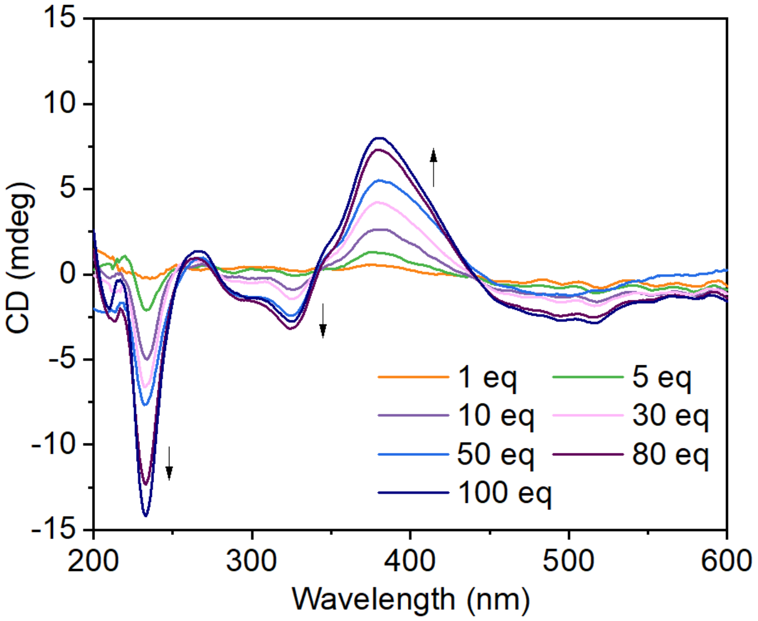

Figure S34. CD spectra of M_6-biased **1** in acetonitrile, prepared with different equivalents of (*R*)-BINOL. The concentration was 1×10^{-5} M.

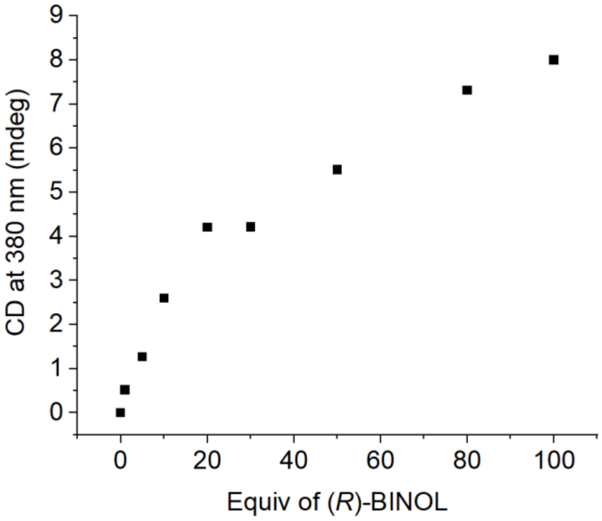

Figure S35. Plot of the relationship between CD intensity and equivalents of BINOL used in the preparation of **1**.

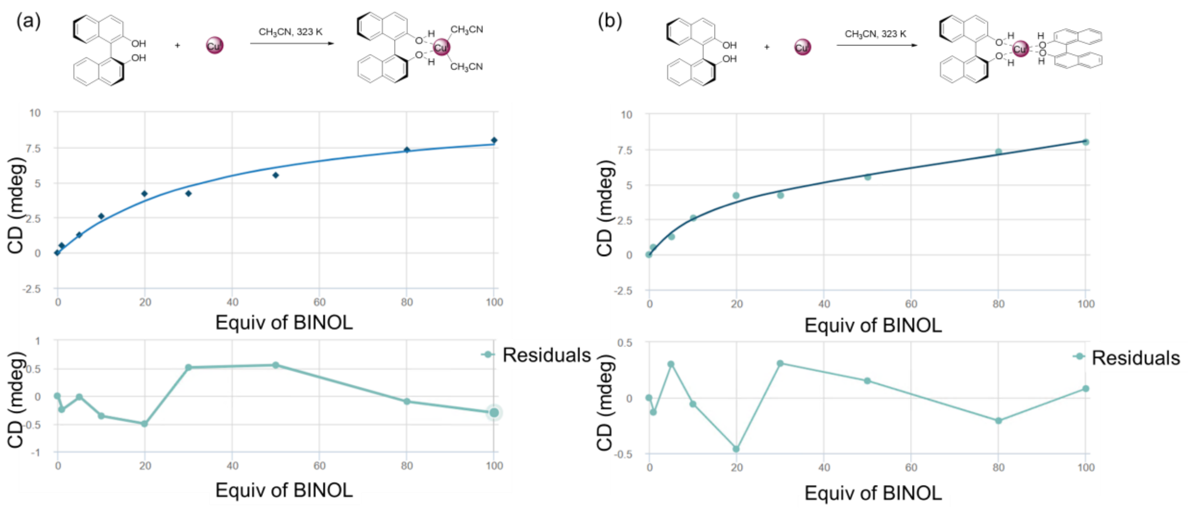

Figure S36. Non-linear curve fitting of the relationship between CD intensity and equivalents of BINOL used during cage synthesis. 1:1 and 1:2 binding models were both chosen for fitting, to investigate the nature of the chiral intermediate formed between Cu^{I} and BINOL that determines the handedness of the dicopper(I) helicate. 1:2 fitting gives better results. As a large excess (up to 100 equiv.) of BINOL was used to induce stereoselectivity, we thus infer that the 1:2 complex-- $Cu^{I}(BINOL)_2$-- is more plausible. The maximum CD intensity is calculated to be 10.5 mdeg. The binding constant here is related to the ability of BINOL to bind Cu^{I} and thus induce the chirality of the cage.

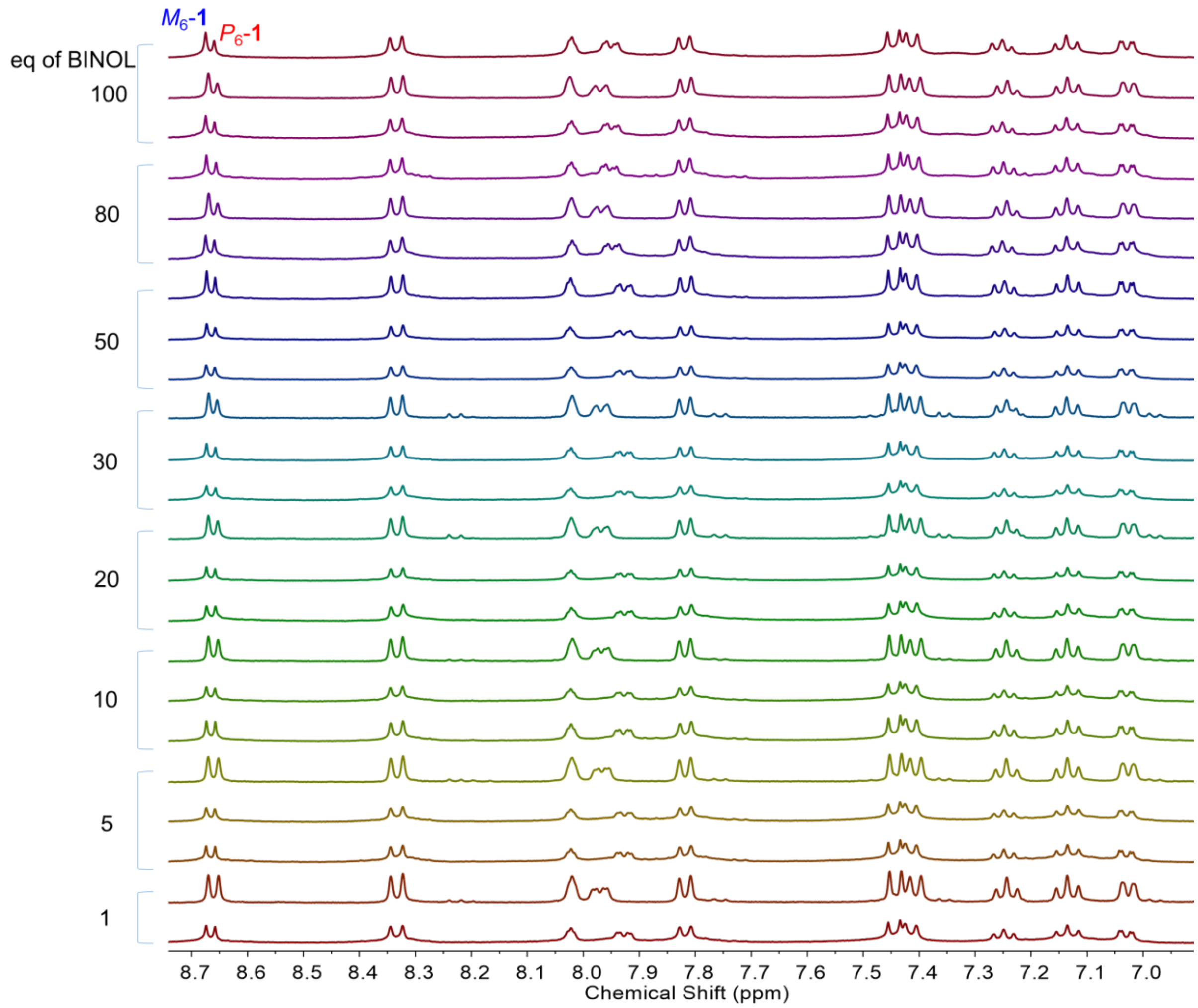

Figure S37. Partial ^{1}H NMR (400MHz, 298 K, CD_3CN) of M_6-biased **1** prepared with different equivalents of (*R*)-BINOL in the presence of 2.00 equiv of *Δ*-TRISPHAT. Each group contains parallel experiments that are marked by brackets.

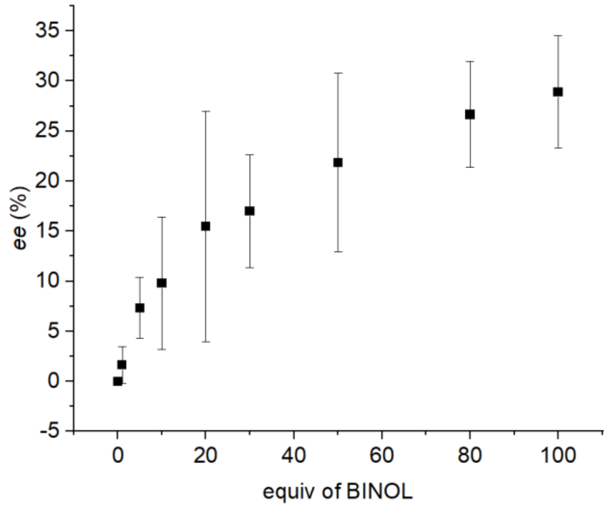

Figure S38. Plot of the relationship between *ee* and equivalents of BINOL used in the synthesis of **1**.

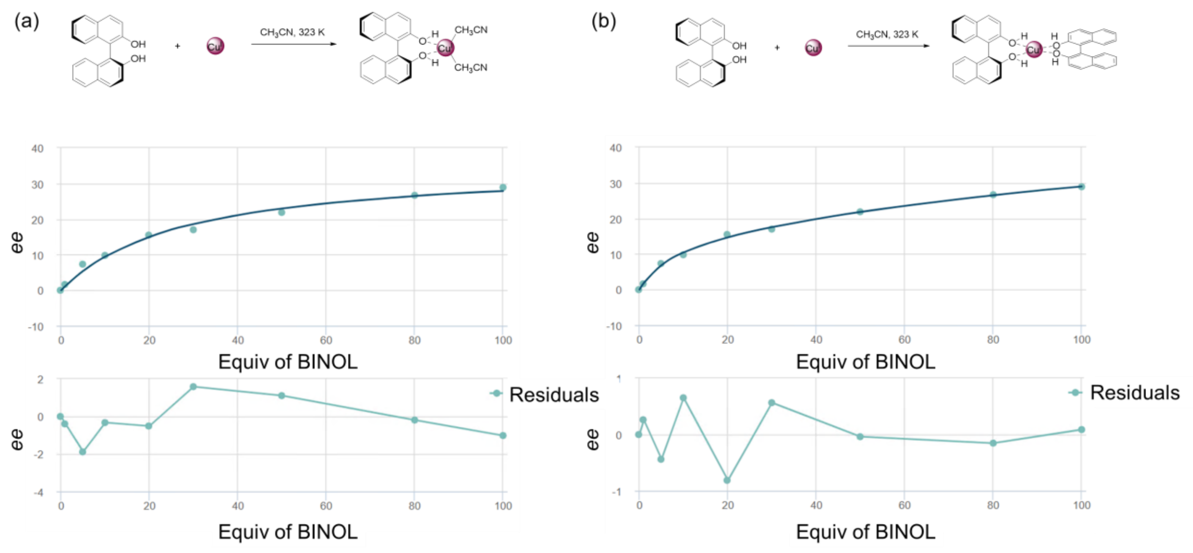

Figure S39. Non-linear curve fitting of the relationship between *ee* and equivalents of BINOL used during cage synthesis. 1:1 and 1:2 binding models were chosen for fitting, to investigate the nature of the chiral intermediate formed between Cu^{I} and BINOL that determines the handedness of the dicopper(I) helicate. The1:2 fitting gives better results and as a large excess of BINOL was used to induce stereoselectivity, we thus infer the 1:2 complex--$Cu^{I}(BINOL)_2$--is the key compound. The maximum *ee* is calculated to be 35.5%. The binding constant here is related to the ability of BINOL to bind Cu^{I} and induce the chirality of **1**.

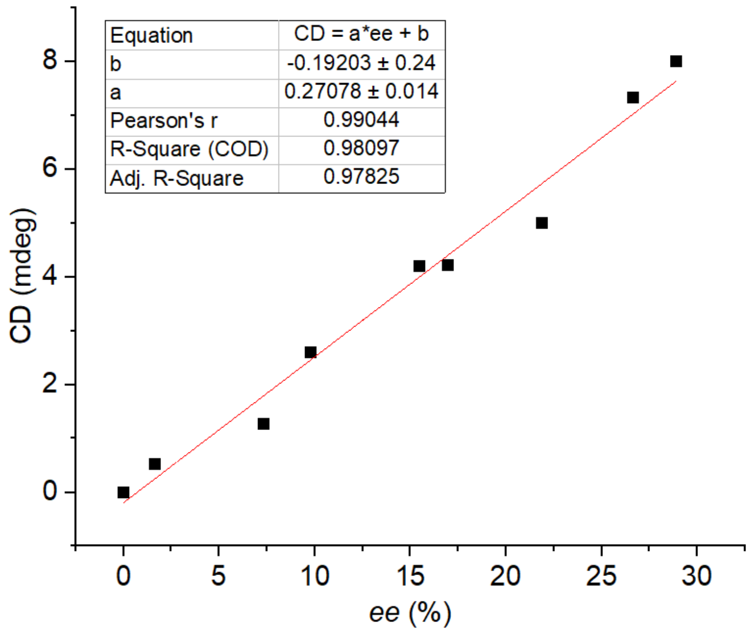

Figure S40. Linear curve fitting of the relationship between CD signal intensity and *ee* in each sample prepared using different equivalents of (*R*)-BINOL. The linear correlation indicates CD signal intensity and *ee* match well with each other.

Proposed mechanism of stereoselectivity

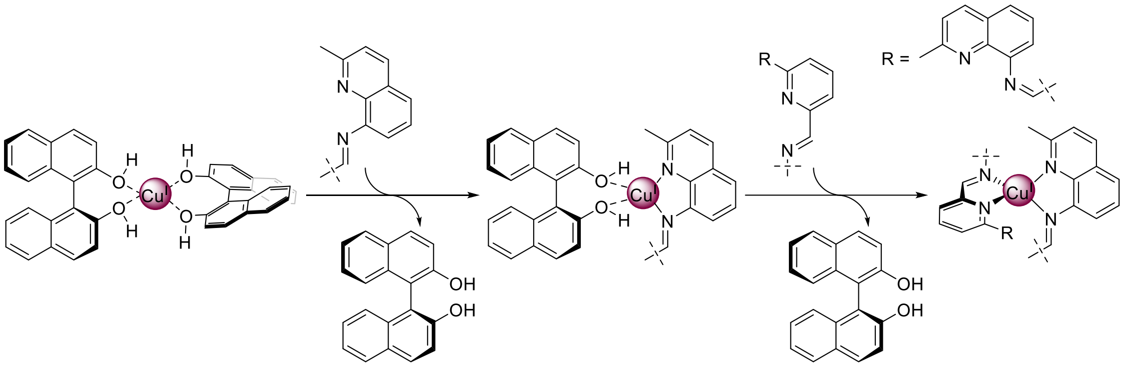

Scheme S4. Proposed mechanism of stereoselectivity.

We infer that the complexation between Cu^I and BINOL generates a chiral intermediate, which is followed by ligand exchange to produce a chiral center that determines the handedness of the helicate. This hypothesis is supported by the following observations:: (i) There is no interaction between BINOL and cage **1** (Figure S41). (ii) The chirality of **1** remains stable without BINOL, and adding (*S*)-BINOL into a solution of M_6-**1** or racemic **1** does not change the chirality of **1**, indicating the chirality is determined at the initial stage of assembly. (iii) Once formed the cage is kinetically stable to racemization implying chiral information during the formation of the cage is crucial. (iv) Adding more BINOL or decreasing the temperature, which are methods to increase the formation of the chiral intermediates, afford higher CD intensity and *ee*. (v) Using chiral additives with too weak or too strong binding didn't yield enriched **1**, as discussed below.

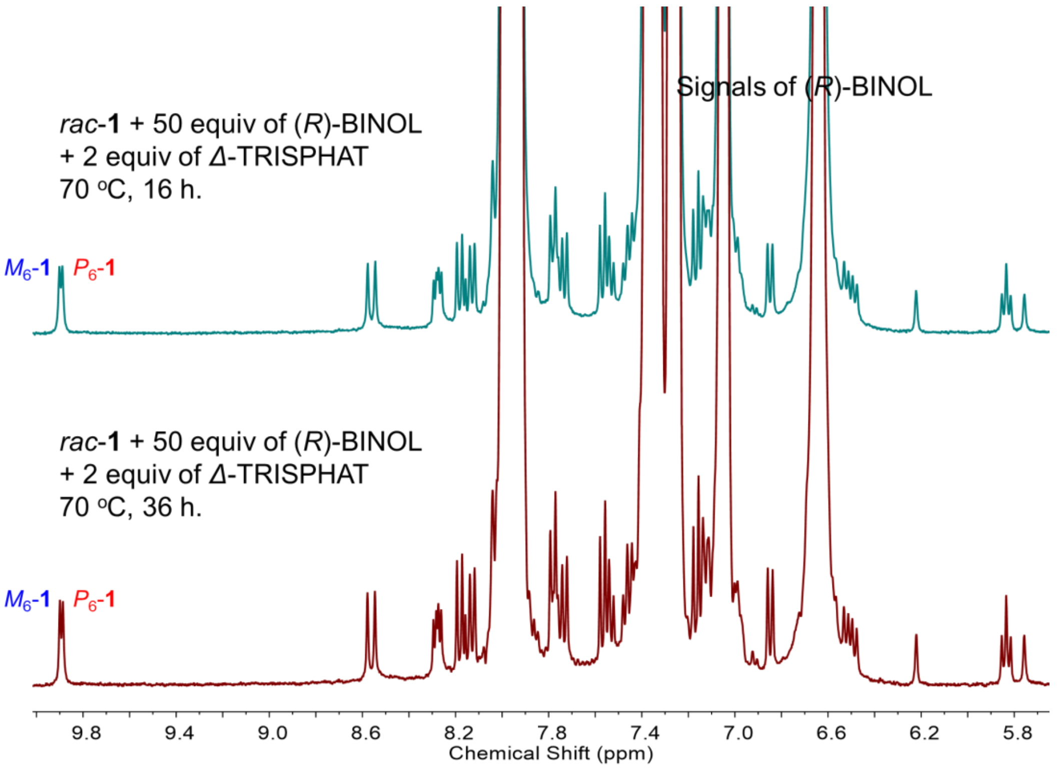

Figure S41. Aromatic region of the ^{1}H NMR spectra (400MHz, 298 K, CD_3CN) of a mixture of **1**, 50 equiv of (*R*)-BINOL and 2 equiv of *Δ*-TRISPHAT after heating at 70 °C for 16 and 36 h. The ratio of the two enantiomers remained unchanged, implying the chirality of the system does not change once the cage forms. This provides evidence for the proposed mechanism that the chirality is determined at the initial stage of assembly.

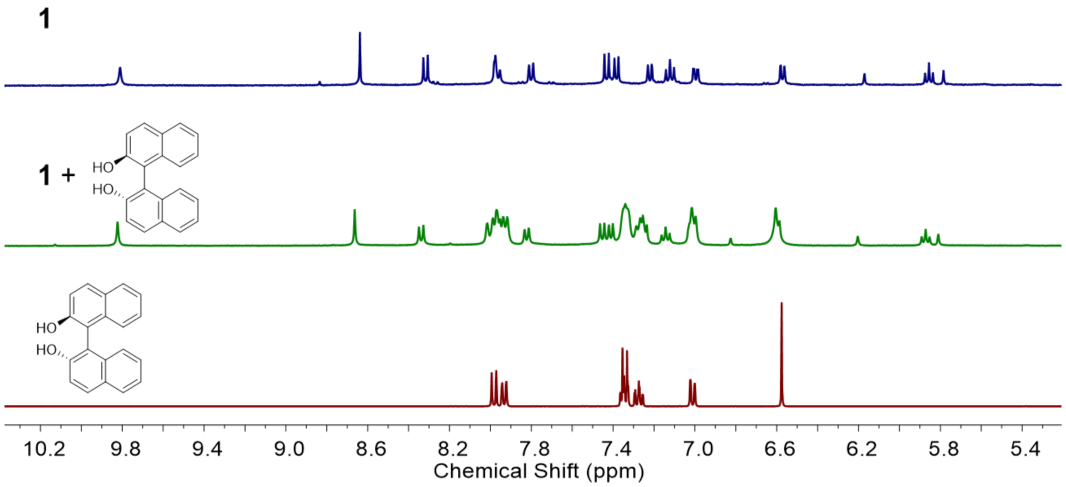

Figure S42. ^{1}H NMR spectra (400MHz, 298 K, CD_3CN) of **1** (top), **1** + BINOL (5 equiv, middle) and BINOL (bottom). No chemical shift changes were observed for **1** after adding BINOL, revealing no interactions between them. Therefore, BINOL is not a guest of **1** and doesn't have peripheral interactions with **1**.

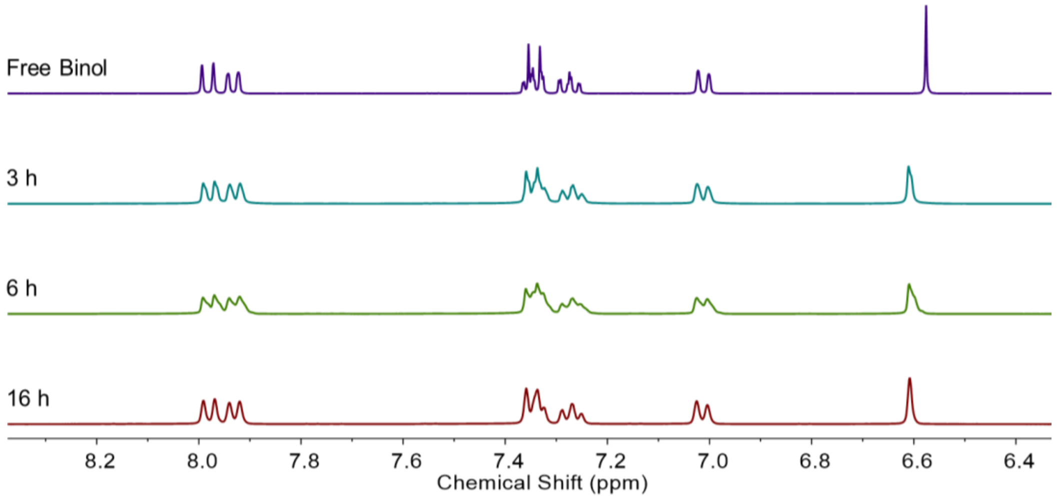

Figure S43. ^{1}H NMR spectra (400MHz, 298 K, CD_3CN) of BINOL before (top) and after adding tetrakis(acetonitrile)copper(I) triflate (1 equiv). The spectra were recorded after 3, 6, and 16 h of heating at 323 K. The downfield chemical shift changes demonstrated complexation.

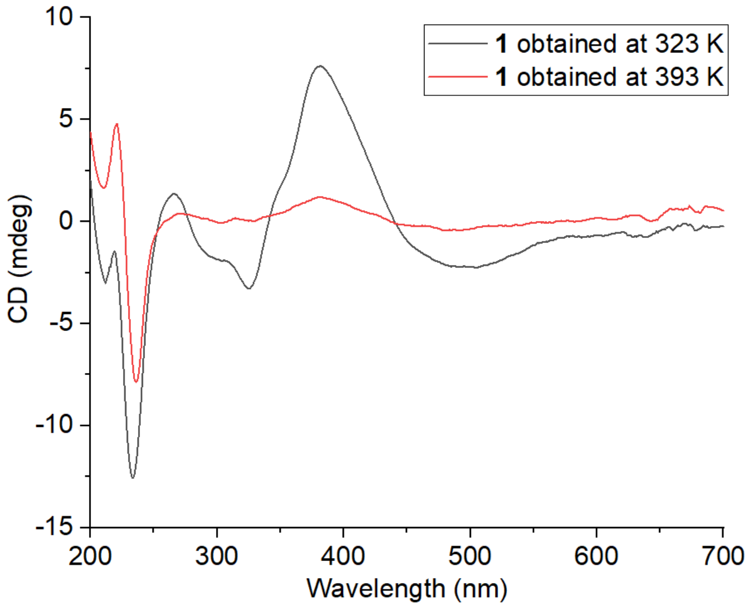

Figure S44. CD spectra of M_6-biased **1** prepared by oil bath heating at 323 K (black line) and microwave heating at 393 K (red line). 50 equiv of (*R*)-BINOL was used for each reaction. The concentration was 1×10^{-5} M. The lower CD intensity of **1** prepared at higher temperature also provides evidence for the formation of the Cu^{I}-BINOL complex that determines the chirality of **1**, because such a complex is expected to be less stable at higher temperature.

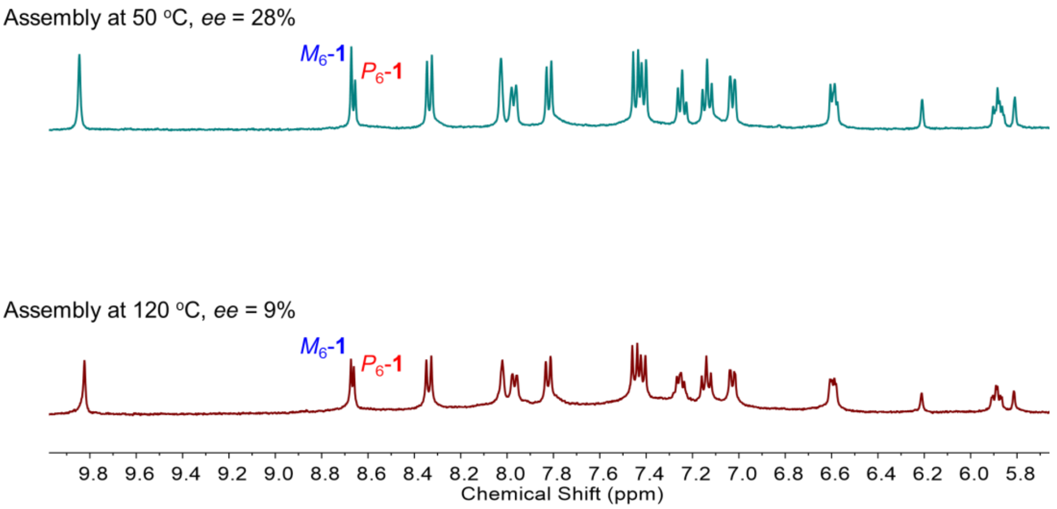

Figure S45. ^{1}H NMR spectra (400MHz, 298 K, CD_3CN) of M_6-biased **1** prepared with 50 equiv of (*R*)-BINOL at different temperatures. 2.00 equiv of *Δ*-TRISPHAT was added for chiral discrimination. A higher *ee* was obtained at lower temperature, consistent with the importance of the proposed Cu^I-BINOL chiral intermediate.

Attempts to induce chirality by other chiral compounds

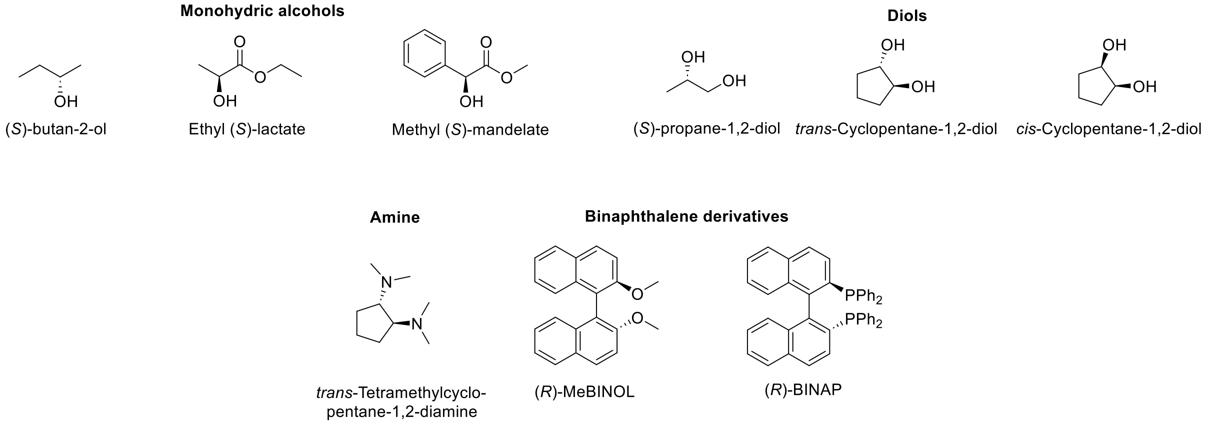

Figure S46. Compounds tested for their ability to induce the chirality of the helicates in **1**.

All compounds in Figure S45 were screened for their ability to induce the chirality of the helicates in **1** by adding 50 equiv of them before assembly. Racemic **1** was obtained in the presence of all monohydric alcohols and diols. The failure of the chiral induction could be ascribed to extremely weak binding between these alcohols and Cu^I. Adding 50 equiv of tetramethylcyclopentanediamine, MeBINOL and BINAP prevented the cage formation, only insoluble precipitates were obtained. We infer that the binding between these three compounds and Cu^I is too strong, whereby subsequent ligand exchange doesn't take place. This

phenomenon also indicates our proposed $Cu^{I}(BINOL)_2$ complex is crucial and BINOL has the most suitable binding affinity to Cu^{I}.

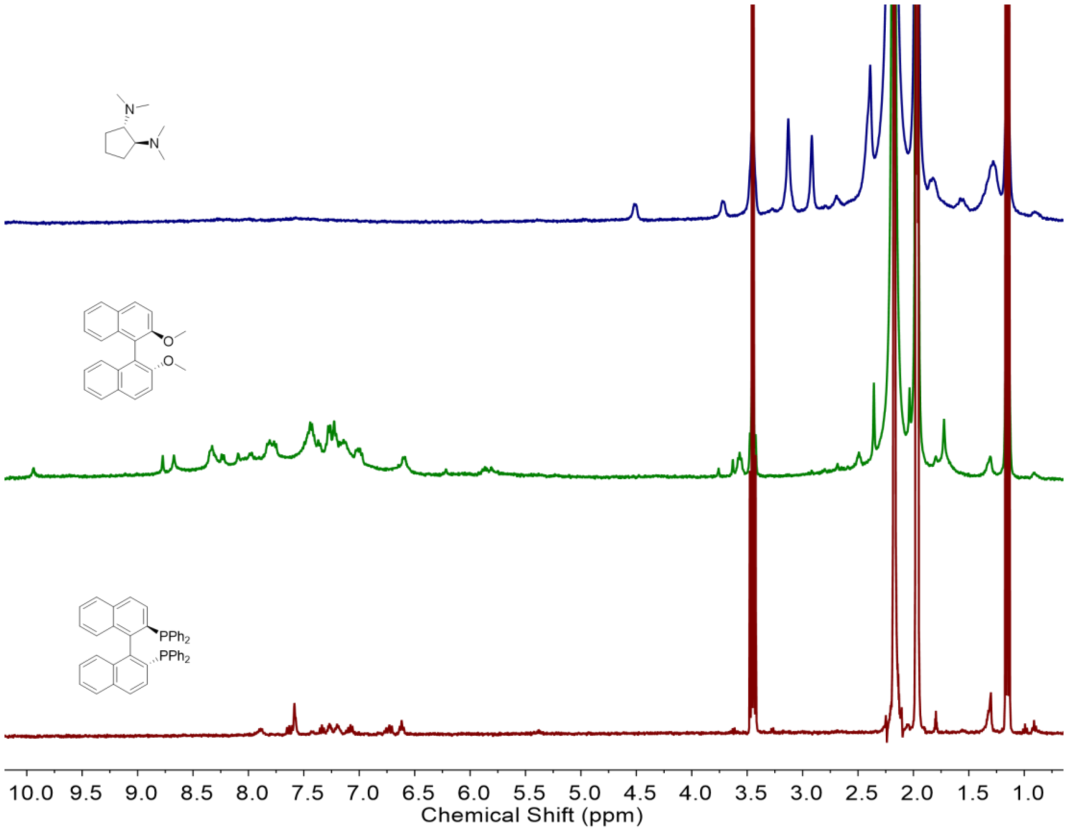

Figure S47. ^{1}H NMR spectra (400MHz, 298 K, CD_3CN) of self-assembly reactions for the attempted preparation of cage **1** in the presence of 50 equiv of different chiral additives. Chiral additives were removed by washing with ethyl acetate or toluene and then diethyl ether before NMR spectra were recorded.

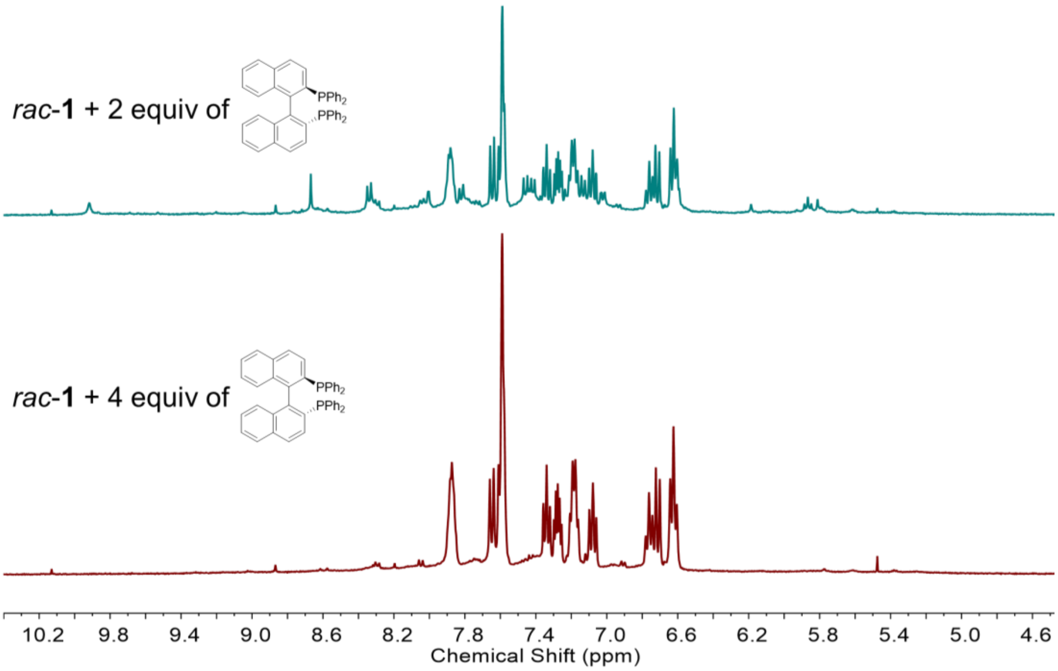

Figure S48. ^{1}H NMR spectra (400MHz, 298 K, CD_3CN) of mixtures of **1** and different equivalents of BINAP upon heating for 16 h. Cage **1** decomposed after heating, indicating the binding affinity between BINAP and Cu^{I} is high.

6. Photophysical studies of **1**

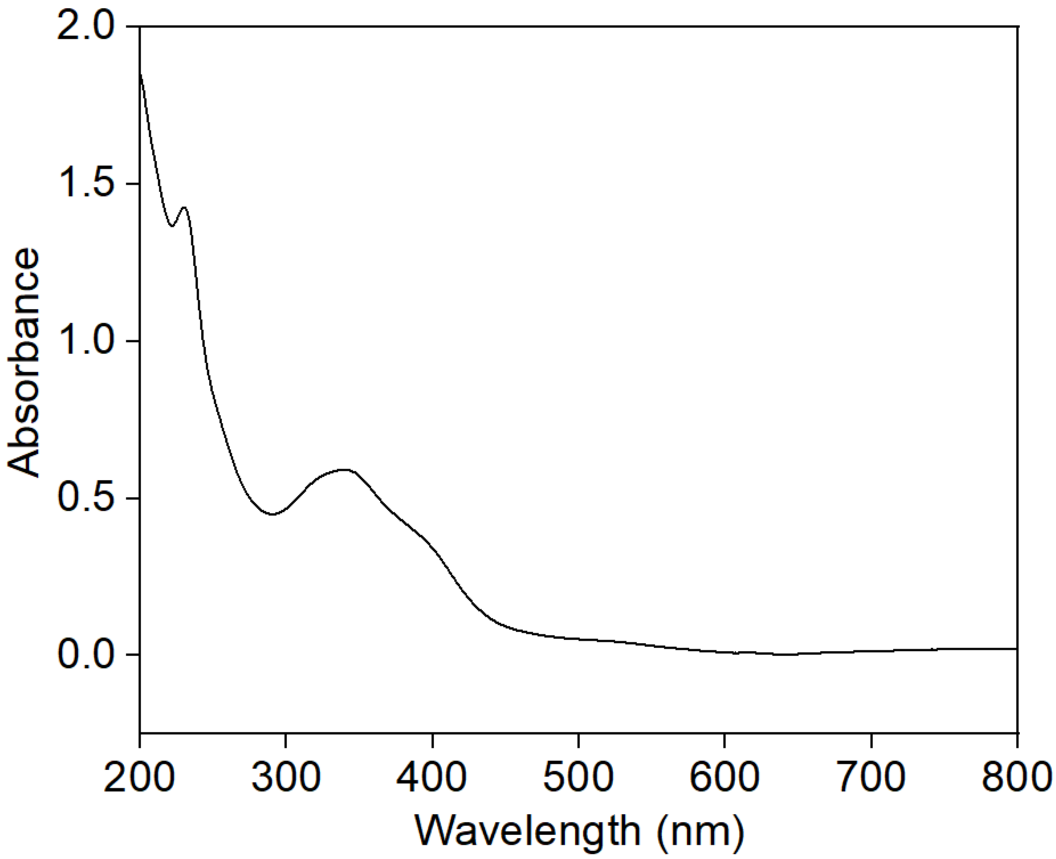

Figure S49. UV-vis spectrum of **1** in acetonitrile (1×10^{-5} M).

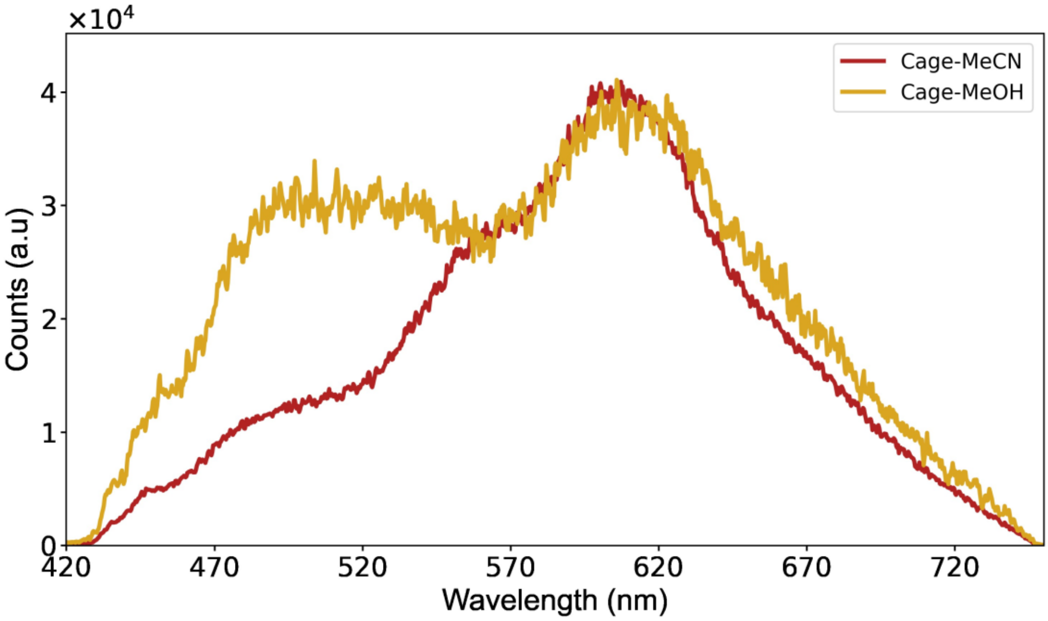

Figure S50. PL spectra of cage **1** in acetonitrile (red) and methanol (yellow). The excitation wavelength is 400 nm. The concentration is 10^{-5} M.

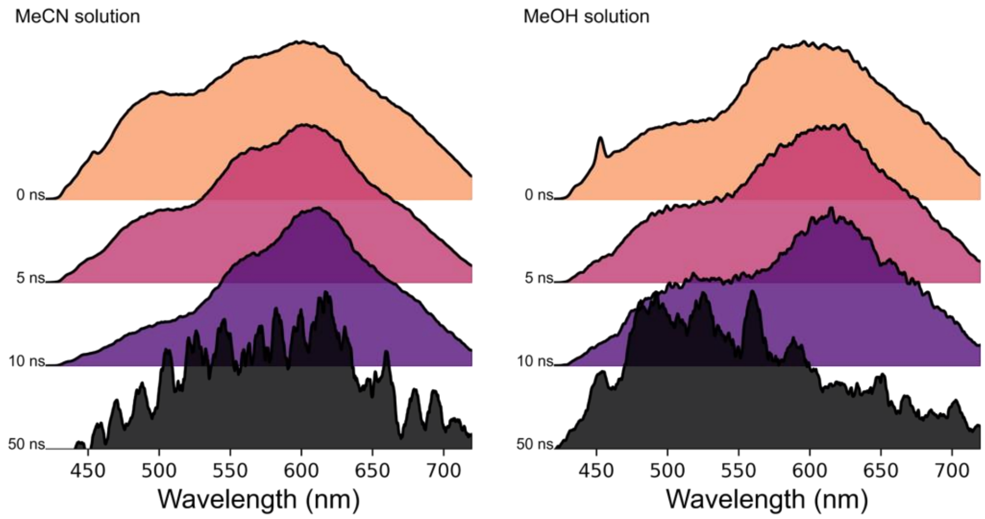

Figure S51. Time-resolved PL spectra of **1** in acetonitrile (left) and methanol (right). All emissions faded in a nanosecond level, indicating singlet-involved radiative decay.

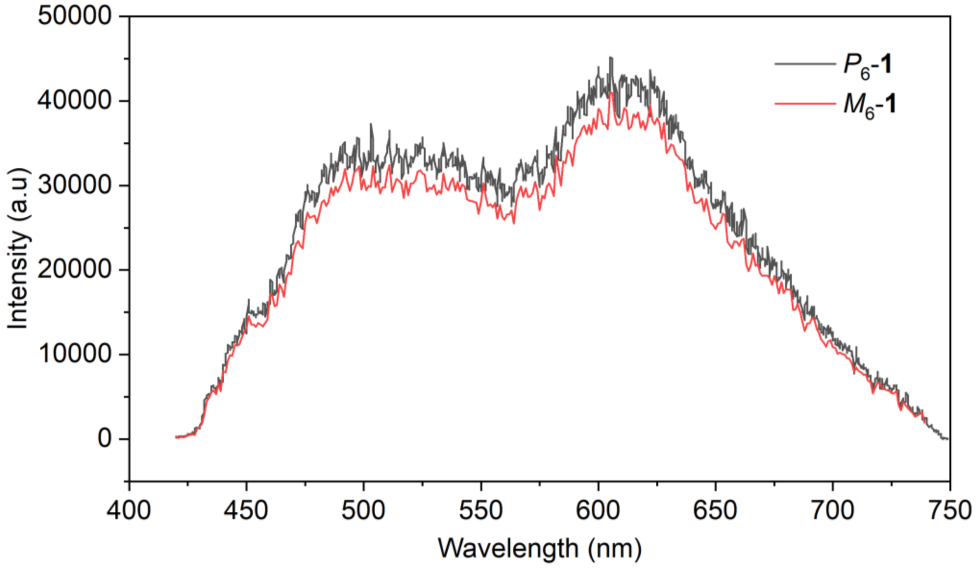

Figure S52. CPPL spectra of P_6- and M_6-**1** in acetonitrile measured by a homemade spectrometer equipped with a rotating quarter waveplate. Photons are captured and thus all signals are positive only. Negative signals can be seen on the g_{lum} spectrum in the manuscript.

7. Computational optimization of the cage structure

Geometrical structure optimization

Every structure was optimised by using Gaussian16 software.[2] The structures were optimized by means of density functional theory via B3LYP functional using 6-31g* basis set.[3] Before performing the geometrical optimization, for each structure the wavefunction was guessed by using the fragments-guess option available on Gaussian16 in order to ease the first self-consistent field (SCF) cycle to converge. Each organic components were assumed to be zero-charged, while each Cu-atoms were assumed to be charged +1e. All the components were assumed to be in singlet state. The total structure was therefore considered with +12e charge in singlet state. The SCF cycles were performed with the XQC algorithm with "tight" convergence option. Each geometry optimization step was performed with the smallest step-size ("maxstep") and the default optimization algorithm and convergence criteria were applied.

Metal-binding domains scan

In order to investigate the possible conformations of the helicates, we isolated the dicopper(I) helicate and investigated the HH and HT conformations, respectively. Anagously to the cage setup, the total charge is +2e and multiplicity is 1. The scan of the conformations were performed starting from the minimum structures, where the distance between Cu-atoms was the shortest at the equilibrium, and then the distances were increased by 0.1 Å each scan step. For each scanned distance the geometrical relaxation was perferfomed restraining the Cu-Cu distance using the "modredundant" option.

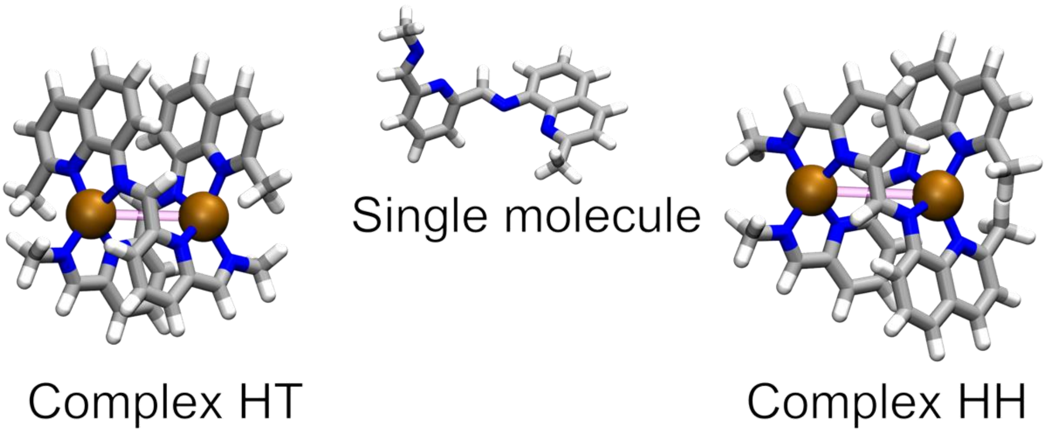

Figure S53. Isolated metal binding domain complexes. The two complexes (HT on the left and HH on the right), investigated at DFT level. The optimal conformations were inquired varying the distance between the two copper atoms (shown in violet).

Generation of the triptycenes geometries

The set of possible triptycene geometries was generated by setting four molecules as they could keep the *T* symmetry. These geometries were scanned varying along two independent modes at the same time. The modes consisted in the distance of the triptycene from the center of the assembly (Figure S47 left) and the rotation the triptycenes around their principal axis (Figure S47 right). Other modes were excluded to maintain the symmetry of the assembly. The distances were sampled every 0.055 Å, while the rotation was sampled every 0.09 deg. A total of 10000 structures were generated. The structures showing overlap or close contact between the molecules were excluded.

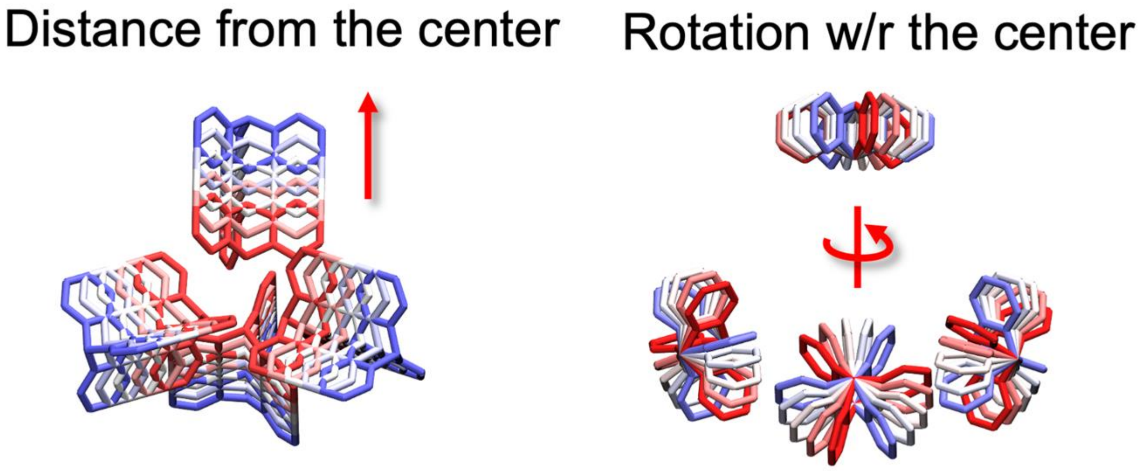

Figure S54. Triptycenes displacements modes. The two modes were used to span the possible conformations. Each color refers to the generated arrangement.

Shape investigation

In order to investigate the possible cage structures that could be assembled given the components, we inquired the possible match that could be observed between the four pre-oriented tripticenes and the metal-binding domains. This match was checked by means of distance, angles and dihedrals formed by the neighbor components. In the cluster binging domain this was expressed as N-N distance (H-H in the pre-oriented tripticenes), CNN angle

(CHH in the pre-oriented tripticenes), and CNNC dihedral (CHHC in the pre-oriented tripticenes). For the pre-oriented tripticenes we considered the inner (figure SXX, left) and outer binding (figure SXX, right).

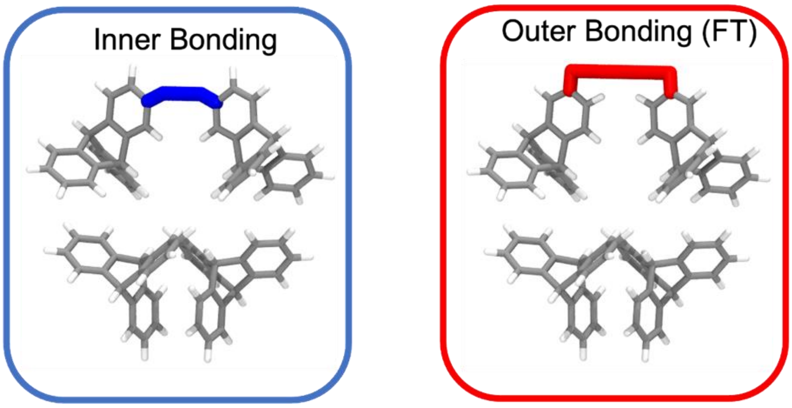

Figure S55. Inner and outer binding of a generic 4 triptycenes assembly.

Representing on a single plot (Figure S49) the distance angle and dihedral that can be observed by the generated structures (inner binding blue colored and outer bonding red colored) along with the values related to the cluster binding domain varying the distance between the two copper atoms, we can observe the possible assemblies of the cage. From this plot we can observe the match between of HH metal binding to form cages with outer bonding and with inner bonding, while for HT it is only possible to form a cage only with inner bonding. The two inner bonding cages lie on the surface of the possible conformations explored in the geometrical study (HT Figure 2a and HH Figure 2b, main text), while the only outer bonding cage (Figure 2c, main text) has some distance from the red-colored region. In the latter case the distance can be justified by the deformations endured by the triptycenes.

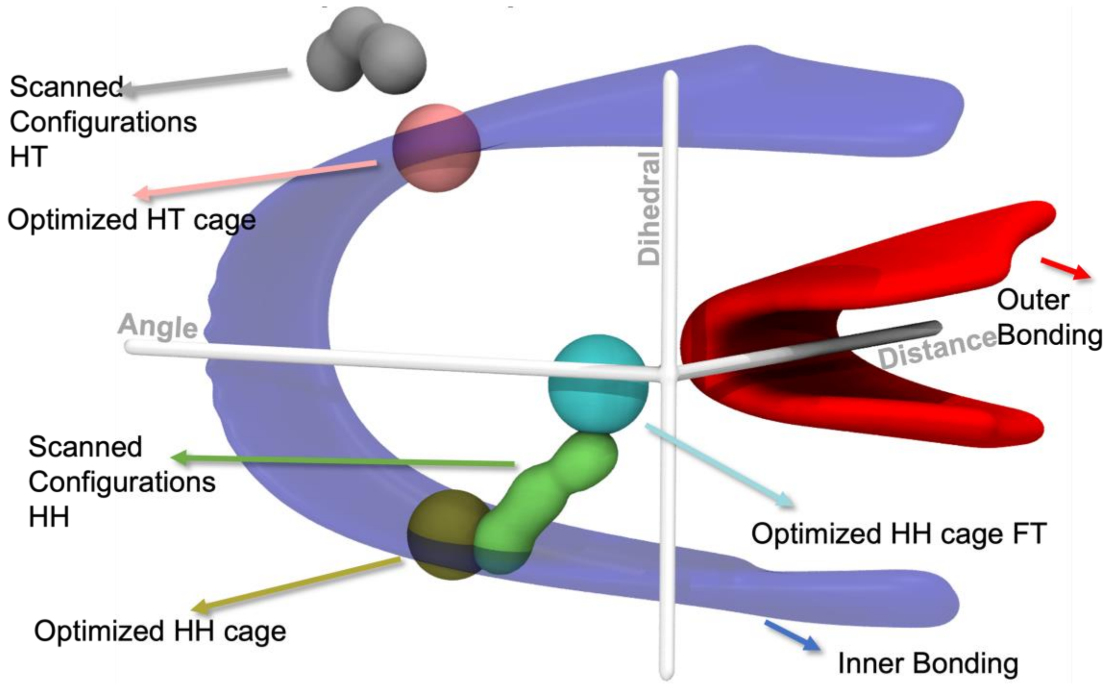

Figure S56. 3D representation of the areas explored by the geometrical investigation (inner binding, blue surface) and inner binding (red surface), along with the scanned configurations of HT (grey colored) and HT (green colored) metal binding observed. The three DFT optimized structures that could be generated. The three coordinates correspond to the distance, angle and dihedral, that should match in order to generate a cage assembly.

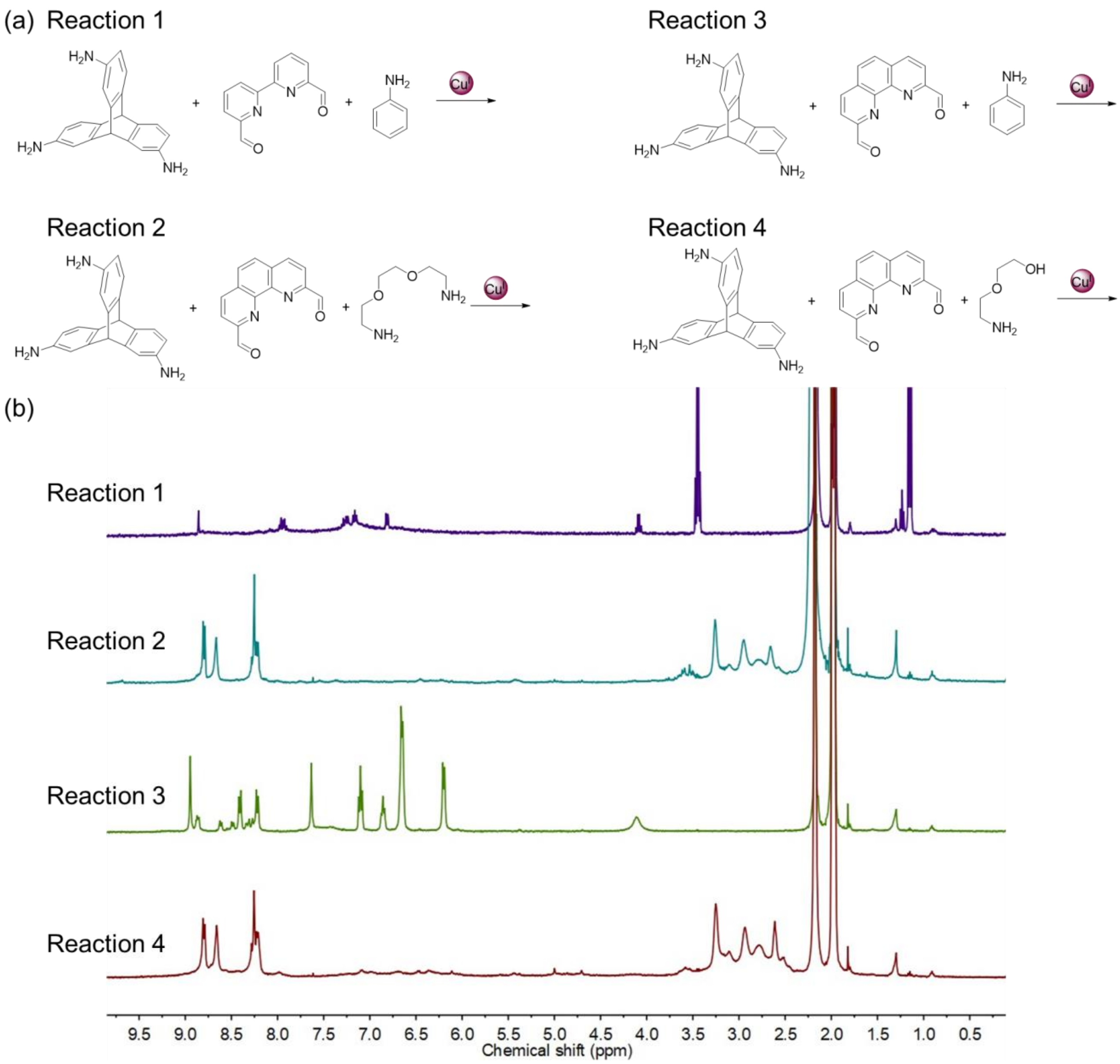

Figure S57. (a) Attempts to prepare helicate-bridged copper(I) cages based on different reactions of triaminotriptycene, diformylbipyridine or diformylphenanthroline, amines and copper(I). (b) ^{1}H NMR spectra (400 MHz, 298 K, CD_3CN) of reactions 1–4 after 18 h at 70 °C. No cage structure was found.

8. References

1. Zhang, C.; Chen, C.-F. *J. Org. Chem.* **2006**, *71*, 6626−6629.

2. Maglic, J. B.; Lavendomme, R. *J. Appl. Cryst*. **2022**, *55*, 1033–1044.

3. Brynn Hibbert, D.; Pall Thordarson, P. *Chem. Commun.*, **2016**, *52*, 12792-12805.

4. Gaussian 16, Revision C.01, M. J. Frisch, G. W. Trucks, H. B. Schlegel, G. E. Scuseria, M. A. Robb, J. R. Cheeseman, G. Scalmani, V. Barone, G. A. Petersson, H. Nakatsuji, X. Li, M. Caricato, A. V. Marenich, J. Bloino, B. G. Janesko, R. Gomperts, B. Mennucci, H. P. Hratchian, J. V. Ortiz, A. F. Izmaylov, J. L. Sonnenberg, D. Williams-Young, F. Ding, F. Lipparini, F. Egidi, J. Goings, B. Peng, A. Petrone, T. Henderson, D. Ranasinghe, V. G. Zakrzewski, J. Gao, N. Rega, G. Zheng, W. Liang, M. Hada, M. Ehara, K. Toyota, R. Fukuda, J. Hasegawa, M. Ishida, T. Nakajima, Y. Honda, O. Kitao, H. Nakai, T. Vreven, K. Throssell, J. A. Montgomery, Jr., J. E. Peralta, F. Ogliaro, M. J. Bearpark, J. J. Heyd, E. N. Brothers, K. N. Kudin, V. N. Staroverov, T. A. Keith, R. Kobayashi, J. Normand, K. Raghavachari, A. P. Rendell, J. C. Burant, S. S. Iyengar, J. Tomasi, M. Cossi, J. M. Millam, M. Klene, C. Adamo, R. Cammi, J. W. Ochterski, R. L. Martin, K. Morokuma, O. Farkas, J. B. Foresman, and D. J. Fox, Gaussian, Inc., Wallingford CT, 2016.

5. Becke, A. D. Density-Functional Thermochemistry. III. The Role of Exact Exchange. *J. Chem. Phys.*, **1993**, *98*, 5648–5652.
